# Oncogenic Pathway Combinations Predict Clinical Prognosis in Gastric Cancer

**DOI:** 10.1371/journal.pgen.1000676

**Published:** 2009-10-02

**Authors:** Chia Huey Ooi, Tatiana Ivanova, Jeanie Wu, Minghui Lee, Iain Beehuat Tan, Jiong Tao, Lindsay Ward, Jun Hao Koo, Veena Gopalakrishnan, Yansong Zhu, Lai Ling Cheng, Julian Lee, Sun Young Rha, Hyun Cheol Chung, Kumaresan Ganesan, Jimmy So, Khee Chee Soo, Dennis Lim, Weng Hoong Chan, Wai Keong Wong, David Bowtell, Khay Guan Yeoh, Heike Grabsch, Alex Boussioutas, Patrick Tan

**Affiliations:** 1 Duke-NUS Graduate Medical School, Singapore; 2 Cellular and Molecular Research, National Cancer Centre, Singapore; 3 Division of Medical Oncology, National Cancer Centre, Singapore; 4 Department of Physiology, Yong Loo Lin School of Medicine, National University of Singapore, Singapore; 5 Section of Pathology and Tumour Biology, Leeds Institute of Molecular Medicine, St. James's University Hospital, Leeds, United Kingdom; 6 Singapore-MIT Alliance, National University of Singapore, Singapore; 7 Department of Internal Medicine, Yonsei Cancer Center, Yonsei University College of Medicine, Seoul, Korea; 8 Department of Surgery, Yong Loo Lin School of Medicine, National University of Singapore, Singapore; 9 Division of Surgical Oncology, National Cancer Centre, Singapore; 10 Department of General Surgery, Singapore General Hospital, Singapore; 11 Cancer Genomics and Biochemistry Laboratory, Peter MacCallum Cancer Centre, East Melbourne, Victoria, Australia; 12 Department of Medicine, Yong Loo Lin School of Medicine, National University of Singapore, Singapore; 13 Department of Medicine (RMH/WH), University of Melbourne, Western Hospital, Footscray, Victoria, Australia; 14 Cancer Science Institute of Singapore, Yong Loo Lin School of Medicine, National University of Singapore, Singapore; 15 Genome Institute of Singapore, Singapore; Cornell University, United States of America

## Abstract

Many solid cancers are known to exhibit a high degree of heterogeneity in their deregulation of different oncogenic pathways. We sought to identify major oncogenic pathways in gastric cancer (GC) with significant relationships to patient survival. Using gene expression signatures, we devised an *in silico* strategy to map patterns of oncogenic pathway activation in 301 primary gastric cancers, the second highest cause of global cancer mortality. We identified three oncogenic pathways (proliferation/stem cell, NF-κB, and Wnt/β-catenin) deregulated in the majority (>70%) of gastric cancers. We functionally validated these pathway predictions in a panel of gastric cancer cell lines. Patient stratification by oncogenic pathway combinations showed reproducible and significant survival differences in multiple cohorts, suggesting that pathway interactions may play an important role in influencing disease behavior. Individual GCs can be successfully taxonomized by oncogenic pathway activity into biologically and clinically relevant subgroups. Predicting pathway activity by expression signatures thus permits the study of multiple cancer-related pathways interacting simultaneously in primary cancers, at a scale not currently achievable by other platforms.

## Author Summary

Gastric cancer is the second leading cause of global cancer mortality. With current treatments, less than a quarter of patients survive longer than five years after surgery. Individual gastric cancers are highly disparate in their cellular characteristics and responses to standard chemotherapeutic drugs, making gastric cancer a complex disease. Pathway based approaches, rather than single gene studies, may help to unravel this complexity. Here, we make use of a computational approach to identify connections between molecular pathways and cancer profiles. In a large scale study of more than 300 patients, we identified subgroups of gastric cancers distinguishable by their patterns of driving molecular pathways. We show that these identified subgroups are clinically relevant in predicting survival duration and may prove useful in guiding the choice of targeted therapies designed to interfere with these molecular pathways. We also identified specific gastric cancer cell lines mirroring these pathway subgroups, which should facilitate the pre-clinical assessment of responses to targeted therapies in each subgroup.

## Introduction

Gastric cancer (GC) is the second leading cause of global cancer mortality [1]. Particularly prevalent in Asia, most GC patients are diagnosed with advanced stage disease [2]. Deregulation of canonical oncogenic pathways such as E2F, K-RAS, p53, and Wnt/β-catenin signaling are known to occur with varying frequencies in GC [3]–[6], indicating that GC is a molecularly heterogeneous disease. Previous studies describing GC diversity in primary tumors have typically focused on single pathways, measuring only one or a few biomarkers per experiment [4],[6],[7]. In contrast, experimental evidence indicates that most cancer phenotypes (uncontrolled growth, resistance to apoptosis, etc) are largely governed not just by single pathways, but complex interactions between multiple pro- and anti-oncogenic signaling circuits [8]. Narrowing this gap between the clinical and experimental arenas will require strategies capable of measuring and relating activity patterns of multiple oncogenic pathways simultaneously in primary tumors.

Previous studies have proposed using gene expression signatures to predict the activity of oncogenic pathways in cancers [9] – here, we hypothesized that patterns of oncogenic pathway activation could be used to develop a genomic taxonomy of GC. Importantly, this pathway-centric strategy differs substantially from previous microarray studies describing expression changes associated with morphological and tissue type differences in GC [10],[11], as pathway signatures (rather than individual genes) are used as the basis for cancer classification. We developed an *in silico* method to map activation levels of different pathways in cohorts of complex primary tumor profiles and validated this pathway-directed classification approach using proof-of-concept examples from breast cancer. We then applied this method to GC to evaluate eleven oncogenic pathways previously implicated in gastric carcinogenesis [3]–[7], [12]–[17]. In total, we analyzed over 300 primary GCs derived from three independent patient cohorts, performing to the best of our knowledge the largest genomic analysis of GC to date. We identified three oncogenic pathways (nuclear factor-κB (NF-κB), Wnt/β-catenin, and proliferation/stem cell) that were deregulated in the vast majority (>70%) of GCs, and functionally validated the pathway predictions *in vitro* using a panel of GC cell lines. Although patient stratification at the level of individual pathways failed to consistently demonstrate significant differences in clinical outcome, patient stratification by oncogenic pathway combinations (e.g. high proliferation/high NF-κB vs. low proliferation/low NF-κB) showed reproducible and significant survival differences in multiple independent patient cohorts, suggesting a critical role for pathway combinations in influencing GC clinical behavior. Our results thus demonstrate that GCs can be successfully taxonomized using oncogenic pathway activity into biologically, functionally, and clinically relevant subtypes.

## Results

### Predicting Pathway Activation in Cancer Gene Expression Profiles

**Figure 1:**
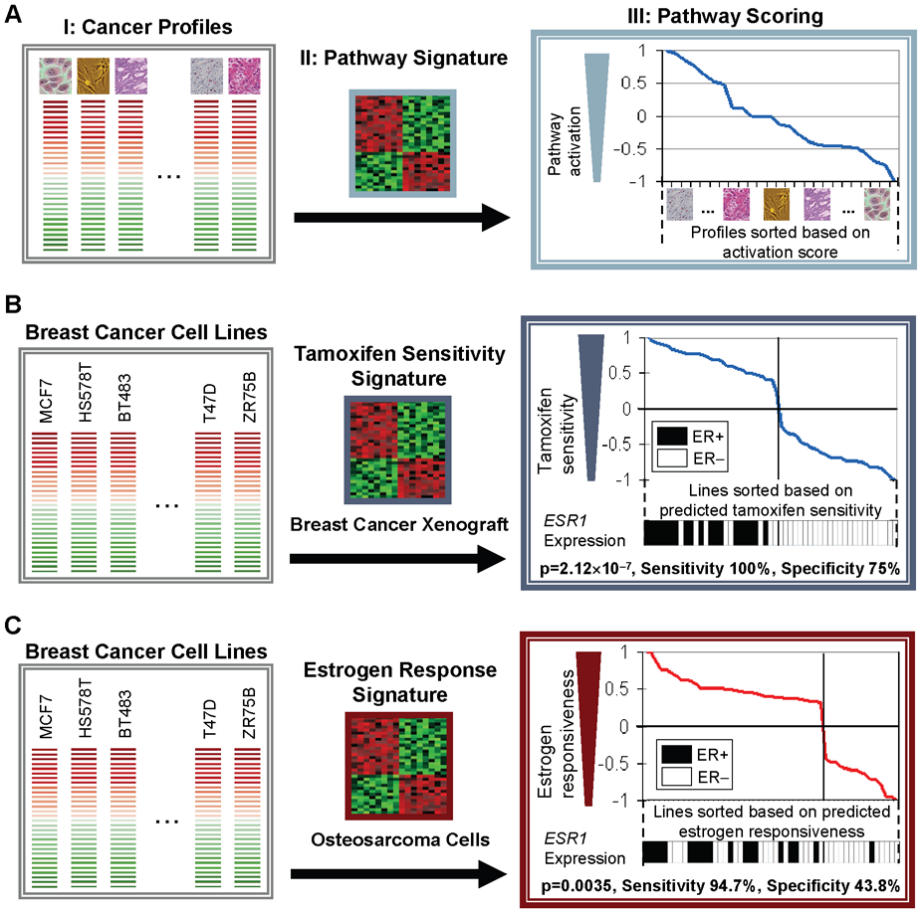
Predicting pathway activation in cancers using gene expression signatures. (A) Schematic of the pathway prediction workflow. I) Expression profiles of a set of cancer samples are pre-processed to identify differentially expressed genes (red and green) compared against a common reference. II) A pathway signature is derived from an independent study concerning the cellular pathway of interest. III) The cancer profiles are compared to the pathway signature using connectivity metrics [37], and subsequently sorted against one another according to the strength of pathway association (pathway scoring). (B) Pathway predictions in breast cancers using a breast-derived tamoxifen sensitivity signature are corroborated by *ESR1* (estrogen receptor) expression, which was used to determine estrogen receptor (ER) status (ER-positive or ER-negative). The cancer profiles are a collection of 51 breast cancer cell lines [18], and the pathway signature generated by comparing a tamoxifen-sensitive mammary xenograft (MaCa 3366) to its tamoxifen-resistant subline (MaCa 3366/TAM) [19]. (C) Pathway predictions in breast cancers using an osteosarcoma-derived estrogen response signature are corroborated by *ESR1* (estrogen receptor) expression. The cancer profiles are a collection of 51 breast cancer cell lines [18], and the pathway signature generated by identifying genes upregulated by estradiol in U2OS osteosarcoma cells [20]. P-values were computed using Pearson's chi-square test, under the null hypothesis that the pathway predictor delivers comparable performance to a random predictor. The *ESR1* gene is absent from both the 11-gene tamoxifen sensitivity signature and the 41-gene estrogen response signature. Only a two-gene overlap exists between both signatures.

Our strategy for predicting levels of oncogenic pathway activation in cancers involves four steps (Figure 1A). First, we defined ‘pathway signatures’ - sets of genes exhibiting altered expression after functional perturbation of a specific pathway in a well-defined *in vitro* or *in vivo* experimental system. Second, we mapped the pathway signatures onto gene expression profiles from a heterogeneous series of cancers. Third, using a nonparametric, rank-based pattern matching procedure, activation scores were assigned to individual cancers based upon the strength of association to the pathway signature. Finally, the individual cancers were sorted based upon their pathway activation scores.

Before applying this approach to GC, we considered it important to validate this *in silico* strategy in a series of proof-of-principle experiments. We chose the example of breast cancer, a malignancy for which there is ample evidence of pathway heterogeneity and discrete ‘molecular subtypes’ [18]. To perform this validation, we first asked if previously described pathway signatures associated with impaired estrogen signaling could be used to identify breast cancer cell lines exhibiting high levels of estrogen receptor (ER) activity. We analyzed a gene expression panel of 51 breast cancer cell lines originally described in Neve at al. [18] with an 11-gene ‘tamoxifen sensitivity’ pathway signature derived from a list of genes differentially expressed between MaCa 3366, a tamoxifen-sensitive human mammary carcinoma xenograft, and MaCa 3366/TAM, a tamoxifen-resistant subline of the same xenograft [19]. We found that breast cancer cell lines positively associated with the tamoxifen sensitivity signature exhibited significantly higher expression levels of *ESR1*, the estrogen receptor and molecular target of tamoxifen, compared to lines showing negative pathway activation scores (p = 2.12×10^−7^, Accuracy 84.3%, Sensitivity 100%, Specificity 75%) (Figure 1B and Table S1).

Second, we tested if a pathway signature associated with estrogen signaling but derived from non-breast tissue could also be used to stratify the same panel of breast cancer cell lines. We queried the breast cancer cell line panel with a 41-gene ‘estrogen response’ signature derived from a list of genes upregulated by estradiol in U2OS human osteosarcoma cells [20]. Despite the signature originating from a different tissue type (e.g. osteosarcoma), we once again found that, when sorted based upon their predicted estrogen responsiveness, breast cancer cell lines clustered together by their level of *ESR1* (estrogen receptor) expression (p = 0.0035, Accuracy 62.7%, Sensitivity 94.7%, Specificity 43.8%) (Figure 1C and Table S1). These results demonstrate that it is indeed feasible to predict patterns of pathway activation in a particular cancer of interest (gastric cancer in our cases) using expression signatures obtained from different experimental conditions and even different tissue types.

### Patterns of Oncogenic Pathway Activation in GC

After validating this pathway prediction approach, we proceeded to apply the strategy to primary GC. Rather than testing every possible pathway, we selected eleven oncogenic and tumor suppressor pathways previously implicated in gastric carcinogenesis, using in our analysis RAS [4], p53 [5], BRCA1 [12], p21 [13], Wnt/β-catenin [6], E2F [3], SRC [14], MYC [15], NF-κB [21], histone deacetylation (HDAC) [16], and stem-cell related signatures [17]. Whenever possible, we attempted to select multiple signatures for each pathway, preferably from independent published studies. For example, of the two E2F activation signatures used in our approach, one signature was obtained by inducing E2F1 activity in rat fibroblast cells [22] while the other signature was obtained using an osteosarcoma-derived cell line containing an inducible ER-E2F1 fusion protein [23]. Final pathway predictions for further analyses were typically obtained by combining individual signatures belonging to the same pathway (see Materials and Methods).

**Figure 2:**
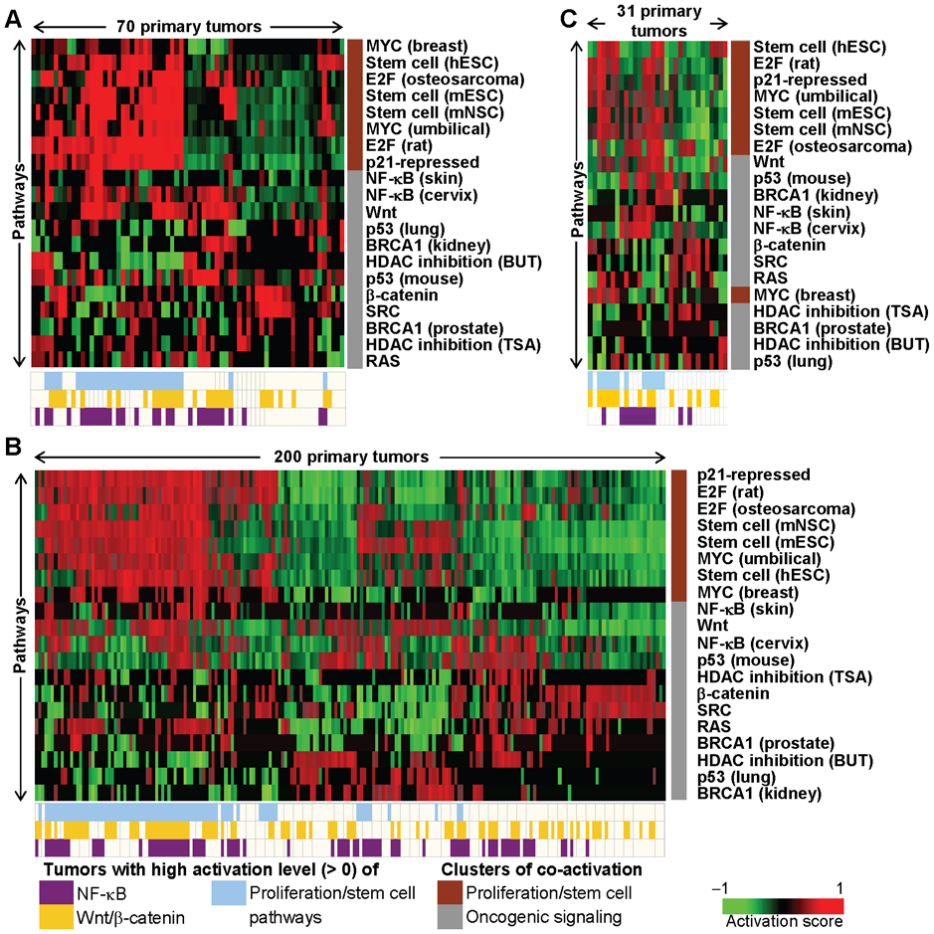
Patterns of pathway activation in primary gastric cancers. Twenty gene expression signatures representing 11 cancer-related pathways (MYC, p21-repression, E2F, NF-κB, RAS, Wnt/β-catenin, SRC, BRCA1, p53, HDAC inhibition, stem cell) were queried against 301 primary gastric cancer gene expression profiles from three independent patient cohorts—(A) Australia, (B) Singapore, and (C) United Kingdom. Each heatmap depicts the activation scores of pathways represented by the signatures (rows) in individual tumors (columns), with red squares denoting higher activation scores. Both pathways and primary tumors were ordered using unsupervised hierarchical clustering. Pathways related to proliferation or stem cell form a distinct cluster (brown) from other pathways (grey). Tumors with high predicted activation of NF-κB (purple), Wnt/β-catenin (yellow), or proliferation/stem cell-related pathways (blue) are indicated by the relevant color bars at the bottom of the heatmaps. Individual signatures that represent similar pathways are differentiated by the wordings within brackets. E.g. Stem cell (hESC): human embryonic stem cell vs. Stem cell (mESC): mouse embryonic stem cell vs. Stem cell (mNSC): mouse neural stem cell; HDAC inhibition (TSA): trichostatin A vs. HDAC inhibition (BUT): butyrate.

We computed activation scores for the eleven pathways represented by 20 pathway signatures across three independent cohorts of primary GCs derived from Australia (Cohort 1–70 tumors), Singapore (Cohort 2–200 tumors), and the United Kingdom (Cohort 3–31 tumors). To visualize patterns of pathway activation, we depicted each cohort as a heatmap, where the heatmap color represents the predicted strength of activation for each pathway in the individual GCs. We observed considerable heterogeneity of pathway activation between individual GC patients (Figure 2A–2C). However, signatures derived from independent studies representing similar pathways frequently yielded similar prediction patterns (e.g. NF-κB (skin) and NF-κB (cervix)), and a chi-square test confirmed a significant level of similarity in the overall patterns of pathway activation between the Australia and Singapore cohorts (p = 0.00038), and between the Australia and UK cohorts (p = 0.00051, see Table S2) suggesting that the GC pathway predictions are not tied to a specific patient cohort. We identified two major clusters of co-activated pathways, which were completely preserved in Cohorts 1 and 2 (Figure 2A and 2B) and mostly preserved in Cohort 3 (Figure 2C). These included (i) a ‘proliferation/stem cell’ pathway cluster (brown vertical bar in Figure 2) encompassing pathways associated with various cell cycle regulators (e.g. MYC, E2F, p21) and stem cell signatures; and (ii) an ‘oncogenic signaling’ pathway cluster (grey vertical bar in Figure 2) containing many different oncogenic pathways (BRCA1, NF-κB, p53, Wnt/β-catenin, SRC, RAS, and HDAC pathways).

### *In Vitro* Validation of Pathway Predictions

By analyzing the GC pathway heatmap in Figure 2, we selected three oncogenic pathways (NF-κB, Wnt/β-catenin, and proliferation/stem cell) that were individually activated in a significant proportion of GCs (≥35%), and when combined provided coverage of the majority (>70%) of GCs. Proliferation/stem cell pathways were activated in 40% of GCs in each cohort (range: 38 to 43%), Wnt/β-catenin pathways were activated in 46% of GCs (range: 43 to 48%), and the NF-κB pathway was activated in 39% of GCs (range: 35 to 41%) (color bars below each heatmap in Figure 2). These frequencies and other frequently deregulated pathways (e.g. p53) are listed in Table S3.

**Figure 3:**
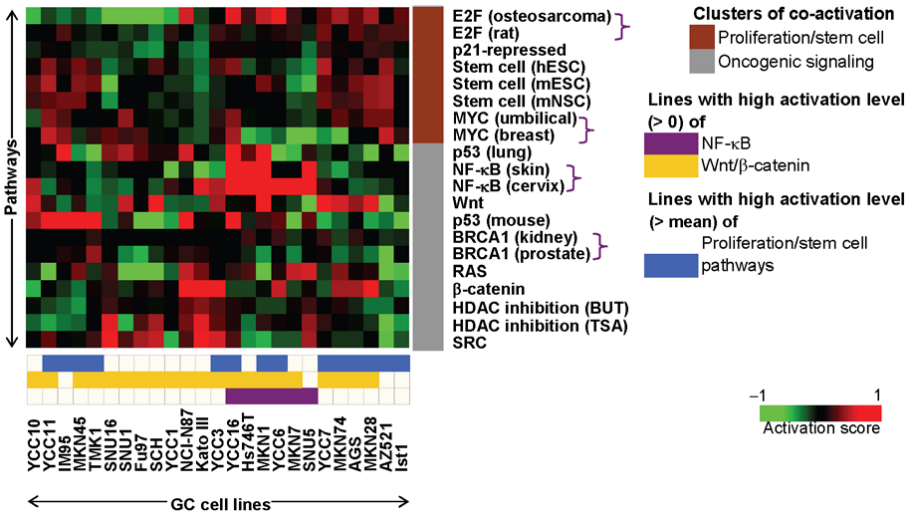
Patterns of pathway activation in gastric cancer cell lines. Twenty gene expression signatures representing 11 cancer-related pathways (previously described in Figure 2) were queried against a panel of 25 gastric cancer cell lines. The heatmap depicts the activation scores of pathways represented by the signatures (rows) in individual cell lines (columns), with red squares denoting higher activation scores. Pathways and cell lines were ordered using unsupervised hierarchical clustering. Similar to primary tumors, pathways related to proliferation or stem cell form a distinct cluster (brown) from other pathways (grey). Cell lines with high predicted activation of NF-κB, Wnt/β-catenin, or proliferation/stem cell-related pathways are indicated by relevant color bars at the bottom of the heatmap. For the proliferation/stem cell-related signatures, the cell lines were mean-normalized relative to one another against the mean activation score, as all cell lines scored positive for proliferation/stem cell-related pathways.

To experimentally validate these primary GC pathway predictions, we applied the pathway prediction algorithm to a panel of 25 GC cell lines (GCCLs) (Figure 3). Similar to primary GC, ‘proliferation/stem cell’ and ‘oncogenic signaling’ pathway clusters were also observed in the GCCLs. Furthermore, signatures representing the same pathway, but obtained from different studies, such as the two independent MYC-derived signatures [9],[24] also clustered together in the GC cell lines after unsupervised hierarchical clustering (purple brackets in Figure 3). Guided by the pathway predictions, we identified specific GC cell lines exhibiting patterns of oncogenic pathway activity mirroring primary GCs. Confidence in the selection of specific cell lines as *in vitro* models was also achieved by repeating the prediction procedure seven times using a variety of reference profiles, ranging from the median GCCL profile to independent profiles such as non-malignant normal stomach profiles (see Materials and Methods and Table S4). Pairwise comparisons confirmed that any two reference profiles were more likely to produce concurring pathway predictions than conflicting predictions (Text S1 and Table S4). Some examples of representative lines include AZ521 and MKN28 cells, which exhibit activation of proliferation/stem cell pathways, YCC3 and AGS cells for Wnt/β-catenin pathways, and MKN1 and SNU5 cells for the NF-κB pathway.

**Figure 4:**
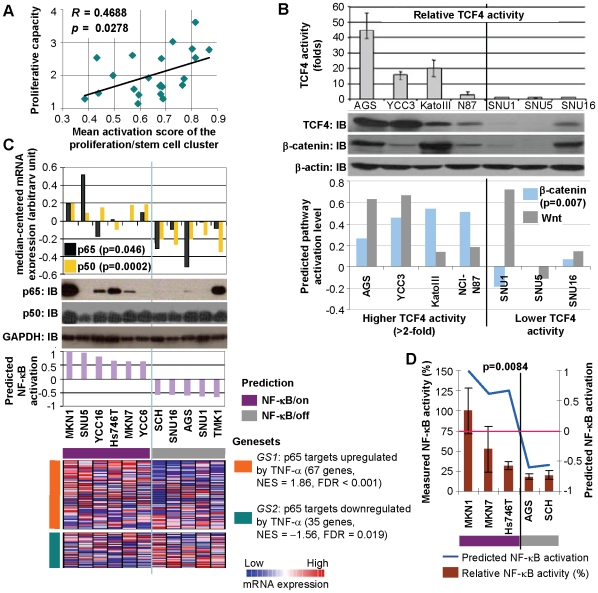
Experimental validation of pathway predictions in gastric cancer cell lines. (A) Experimental validation of proliferation/stem cell pathway predictions. The graph depicts the experimentally measured proliferative capacities of 22 cell lines (y-axis) against the mean proliferation/stem cell activation scores derived from signatures belonging to the proliferation/stem cell cluster. (B) Experimental validation of Wnt/β-catenin pathway predictions. The bottom graph shows the predicted activation levels of the Wnt (grey bars) and β-catenin (blue bars) pathways across seven cell lines. Lines predicted to be active exhibit expression of canonical Wnt pathway components β-catenin and TCF4 (aka TCF7L2) (middle immunoblot), and higher TCF4 transcriptional activity (top graph) compared to lines associated with inconsistent or low Wnt/β-catenin activation scores. Immunoblots were normalized using a β-actin antibody. Parts of this figure were previously presented [50] for a different purpose. (C,D) Experimental validation of NF-κB pathway predictions. (C) The bottom graph shows predicted NF-κB activation levels across 11 cell lines. Lines predicted to be active (‘NF-κB/on’) exhibit significantly higher p65 and p50 mRNA expression levels (topmost graph) and p65 protein expression (immunoblot) relative to lines predicted to be nonactivated (‘NF-κB/off’). All lines exhibit comparable p50 protein expression. Immunoblots were normalized using a GAPDH antibody. Whether p65 target genes are over- or under-expressed in ‘NF-κB/on’ lines compared to ‘NF-κB/off’ lines depends on whether they were up- or downregulated by TNF-α [26], an inducer of NF-κB activation (bottom heatmap). (D) NF-κB activity in cell lines. ‘NF-κB/on’ lines exhibit significantly higher NF-κB transcriptional activity compared to ‘NF-κB/off’ lines.

First, we directly measured the proliferative rates of 22 GCCLs and correlated the proliferation rate data with the mean activation score from signatures in the proliferation/stem cell pathway cluster. There was a significant association between the experimentally determined proliferative rates and the pathway activation scores (R = 0.4688, p = 0.0278) (Figure 4A). Supporting the notion that oncogenic pathway signatures are superior predictors of pathway activity compared to the expression of single key pathway genes, no significant associations were observed for either MYC or E2F1 expression (p = 0.48 and 0.38 for MYC and E2F1, respectively) (Figure S1).

Second, in order to validate the Wnt/β-catenin pathway predictions, we analyzed the expression of various Wnt pathway components (β-catenin, TCF4) and relative levels of TCF/LEF transcriptional activity in GC cell lines predicted to be Wnt/β-catenin- activated or Wnt/β-catenin-nonactivated. Of seven cell lines selected for their experimental tractability (e.g. ease of transfection and convenient growth conditions), we found that both β-catenin and the TCF/LEF transcription factor TCF4 (also known as TCF7L2), major components of the Wnt signaling pathway, were expressed in GC cell lines predicted by the pathway activation analyses to have high Wnt/β-catenin activity (AGS, YCC3, Kato III, and NCI-N87), but not expressed in two out of three lines (SNU1 and SNU5) associated with inconsistent or low Wnt/β-catenin activation scores (Figure 4B). Furthermore, in order to directly assay Wnt pathway activity, we determined TCF/LEF transcriptional activity in the GC cell lines using Topflash, a luciferase expressing plasmid containing multimerized TCF binding sites. The Topflash assay confirmed high TCF/LEF transcriptional activity in three out of four GC cell lines predicted to have high Wnt/β-catenin activity (AGS, YCC3, and Kato III), but minimal or no Topflash activity in GC cell lines associated with inconsistent or low Wnt/β-catenin activation scores (SNU1, SNU5, and SNU16). Additionally, the β-catenin pathway activation scores were significantly higher in GCCLs with more than two-fold TCF/LEF transcriptional activity (AGS, YCC3, Kato III, and NCI-N87) than in GCCLs with lower TCF/LEF transcriptional activity (p = 0.007, Figure 4B). When compared against single genes, superior associations to TCF/LEF transcriptional activity were once again observed using the mean activation score from Wnt/β-catenin signatures compared to either β-catenin or TCF4 (aka TCF7L2) expression alone (p = 0.038 for signatures vs. p = 0.31 and 0.58 for β-catenin and TCF4, respectively) (Figure S1).

Third, to validate the NF-κB pathway predictions, we selected 11 GCCLs consistently predicted as either NF-κB-activated (‘NF-κB/on’, six GCCLs) or NF-κB-nonactivated (‘NF-κB/off’, five GCCLs) (Figure S2). Increased gene expression of p50 and p65, the NF-κB heterodimer subunits, were observed in NF-κB/on GC cell lines compared to NF-κB/off GC cell lines (p = 0.0002 for p50, p = 0.046 for p65, Figure 4C) and at the protein level p65 expression was observed largely in the NF-κB/on lines (Figure 4C). Using immunocytochemistry on formalin fixed paraffin embedded GC cell lines, p65 protein expression was more frequently observed in NF-κB/on GC cell lines compared to NF-κB/off GC cell lines in terms of nuclear sublocalization, percentages of cells with staining (either nuclear or cytoplasmic), and staining intensity (Table S5, Figure S3). To determine if NF-κB/on GC cell lines also exhibited differential expression of p65-regulated genes compared to NF-κB/off GC cell lines, we combined the list of genes directly bound by the p65 transcription factor [25] with lists of genes regulated at the mRNA level by TNF-α [26], a known inducer of NF-κB activation. Using Gene Set Enrichment Analysis (GSEA, [27]), we found that p65 target genes upregulated by TNF-α treatment were significantly overexpressed in NF-κB/on GC cell lines compared to NF-κB/off GC cell lines (normalized enrichment score, NES = 1.86; false discovery rate, FDR<0.001, bottom most panel, Figure 4C). Conversely, p65 target genes downregulated by TNF-α were significantly underexpressed in NF-κB/on GC cell lines compared to NF-κB/off GC cell lines (NES = −1.56, FDR = 0.019, bottom most panel, Figure 4C). Finally, to directly confirm the presence of elevated NF-κB activity, we transfected three NF-κB/on GC cell lines and two NF-κB/off GC cell lines with a luciferase reporter containing a NF-κB reporter gene. As shown in Figure 4D, the three NF-κB/on GC cell lines exhibited elevated NF-κB transcriptional activity compared to the two NF-κB/off GC cell lines (p = 0.0084).

Taken collectively, these results support the concept that *in silico* pathway predictions using gene expression profiles are associated with activation of the relevant pathway *in vitro*.

### Pathway Combinations Predict Gastric Cancer Patient Survival

**Figure 5:**
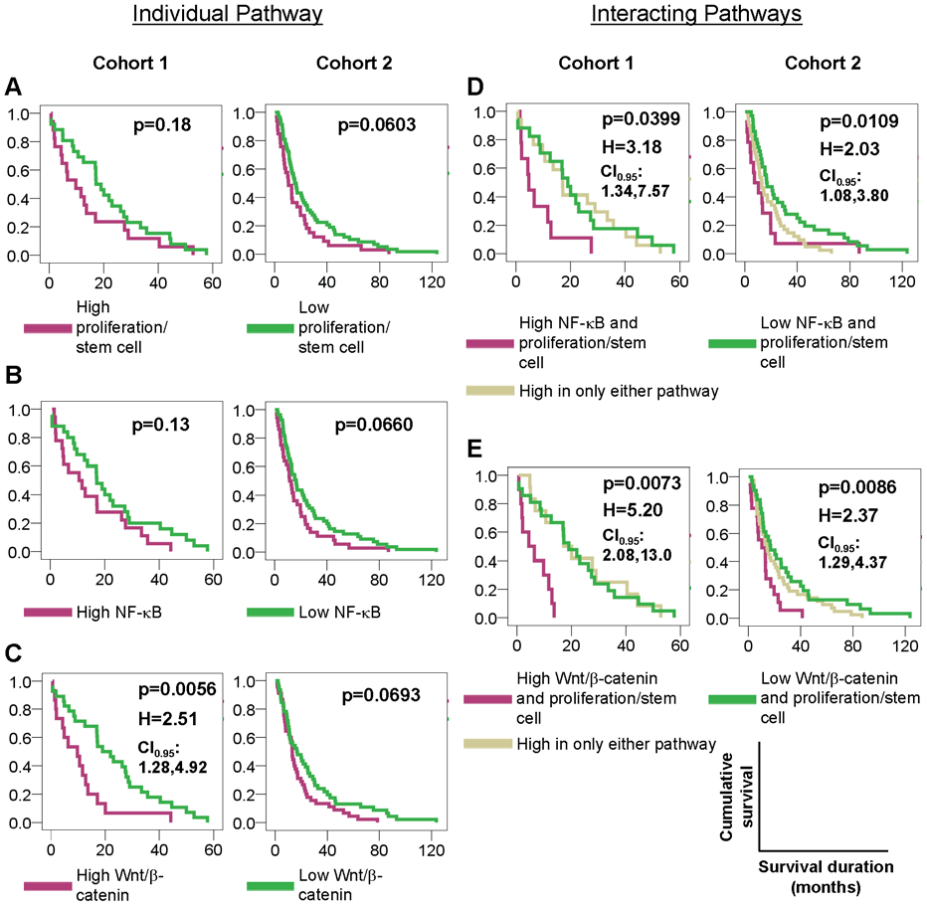
Pathway interactions influence patient survival in gastric cancer. Kaplan-Meier survival analysis of Australia and Singapore cohorts (Heatmaps A and B in Figure 2) between patient groups stratified by predicted pathway activation status. Cohort 3 was not included in the survival analysis as it is much smaller than Cohorts 1 and 2 (31 tumors compared to 70 and 200), making it unreliable for statistical analysis. (A–C) Effects of individual pathways. Patients were stratified by (A) proliferation/stem cell signatures alone, (B) NF-κB signatures alone, and (C) Wnt/β-catenin signatures alone. (D) and (E) Effects of pathway interactions. Patients were stratified by (D) NF-κB and proliferation/stem cell signatures, and (E) Wnt/β-catenin and proliferation/stem cell signatures. For both the NF-κB and Wnt/β-catenin signatures, the significance of the survival difference or death hazard was markedly enhanced by the addition of pathway prediction information from the proliferation/stem cell signatures. The outcome metric was duration of overall survival. H: death hazard indicating the ratio of the mortality rate of patients showing high activation level of single pathway (or both of two pathways) to the mortality rate of patients showing low activation level of single pathway (or both of two pathways). All death hazard ratios are significant at p<0.01. CI_0.95_: 95% confidence intervals for death hazard ratio.

To assess the clinical relevance of the identified pathway subgroups, we investigated if patterns of pathway co-activation as illustrated in the heatmaps of the different cohorts might be related to patient survival. We used overall survival data from Cohort 1 and Cohort 2 and stratified patients by their predicted patterns of pathway activation. A primary GC profile was defined as showing high activation level of a pathway when the activation score was above zero – i.e. being positively associated with the pathway signature. Patient groups stratified by either the proliferation/stem cell pathway activation score alone or the NF-κB pathway activation score alone did not differ significantly regarding their overall survival (p>0.05 for proliferation/stem cell and NF-κB in both cohorts, Figure 5A and 5B). However, when the pathway activation scores were combined, patients with high activation levels of both NF-κB and proliferation/stem cell pathways had significantly shorter survival compared to patients with low activation levels of both NF-κB and proliferation/stem cell pathways (p = 0.0399 and p = 0.0109 for Cohorts 1 and 2 respectively, Figure 5D).

Activation of the Wnt/β-catenin pathway was significantly associated with patient survival in Cohort 1, (p = 0.0056, Figure 5C) but not in Cohort 2 (p = 0.0693, Figure 5C). However, patients in Cohorts 1 and 2 with high activation levels of both Wnt/β-catenin and proliferation/stem cell pathways had significantly worse survival compared to patients with low activation levels of both pathways (p = 0.0073 and p = 0.0086, Figure 5E). To benchmark the contributions of the pathway combinations against known histopathologic criteria, we performed a multivariate analysis including combined pathway predictions and pathological tumor stage (TNM classification: stages 1–4), the most important prognostic factor in GC [28]. In both cohorts, combined activation of proliferation/stem cell and NF-κB pathways proved to be a prognostic factor independent from tumor stage (p = 0.003 and 0.048 for Cohorts 1 and 2, respectively) (Table S6). Likewise, combined activation of proliferation/stem cell and Wnt/β-catenin pathways was an independent prognostic factor in Cohort 1 and achieved borderline significance in Cohort 2 (p<0.001 and p = 0.058, Table S7). These results demonstrate that the assessment of the combined pathway activation status is clinically relevant and moreover can provide additional prognostic information over and above the current gold standard of patient prognosis prediction, the TNM based tumor staging.

## Discussion

In this study, we sought to subdivide GCs into molecularly homogenous subgroups as a first step to individualizing patient treatments and improving outcomes. Importantly, unlike previous GC microarray studies relating gene expression patterns to histology or anatomical type [10],[11], we chose to base our GC subdivisions on patterns of oncogenic pathway activity. After developing and validating this novel classification approach, we were able to describe, for the first time, a genomic taxonomy of GC based on patterns of oncogenic pathway activity. Our approach is particularly suited for gene expression microarrays, since these platforms interrogate thousands of mRNA transcripts in each sample, thereby permitting the assessment of multiple pathways simultaneously in a single experiment. In contrast, such an approach is not currently possible at the protein level due to lack of appropriate platforms. Using this strategy, we identified three dominant pathways showing activation in the majority (>70%) of GCs: proliferation/stem cell, Wnt/β-catenin, and NF-κB signaling.

The ability to perform such “high-throughput pathway profiling” opens many interesting avenues. For example, several studies have previously reported inconsistent results regarding the prognostic impact of different oncogenic pathways in GC - the prognostic implications of proliferation-related antigens such as Ki-67 in GC are not firmly established [29], and high NF-κB activation in GC has been associated with both good and bad GC patient outcome in different studies [7],[30]. It is quite possible that some of this inconsistency may have been due to a historical focus on using conventional methods and analyzing either single pathways or individual pathway components (genes/proteins). Our observation that pathway combinations are predictive of patient outcome suggests that pathway combinations, rather than single pathways alone, may play a critical role in influencing tumor behavior.

Another benefit of high-throughput pathway profiling is the ability to define higher order relationships between distinct oncogenic pathways. In the current study, we consistently observed concomitant activation of E2F, MYC, p21(-repression), and stem cell pathways in tumors (the ‘proliferation/stem cell’ pathway cluster). This is most likely due to increased cellular proliferation in tumor cells, as E2F is important in cell proliferation control and MYC is both a p21-repressor and inducer of cyclin D2 and cyclin-dependent kinase binding protein CksHs2 [31]. Furthermore, stem cells, particularly embryonic stem cells (ESCs), are also known to exhibit high cell proliferation rates [32]. More intriguingly, we also observed close associations between apparently functionally different pathways, such as β-catenin and SRC, as well as HDAC inhibition and BRCA1. Such pathway co-activation patterns may suggest functional interactions between these pathways, which deserve to be studied further. For example, it is possible that activated c-SRC may enhance the expression of the Wnt signaling pathway [33]. Exploring the relationships between pathways showing co-activation may thus provide valuable information regarding the ability of the cancer cell to coordinate the activity of multiple pathways.

A third benefit of the pathway profiling approach is that it facilitates identification of major disease-related pathways. Of the pathways analyzed in this study, the finding that NF-κB signaling may be elevated in a significant proportion of GCs deserves some attention as this pathway has been relatively less explored in GC. Interestingly, while we observed a significant difference in both p50 and p65 (the NF-κB subunits) gene expression between NF-κB/on and NF-κB/off GCCLs, we did not observe overt differential p50 protein expression in these lines, in contrast to p65 (Figure 4C). This may be due to a combination of three reasons. First, the absolute range of p65 gene expression across the cell lines is markedly greater than the absolute range of p50 gene expression (>3×, Figure S4). Second, the Western blotting assay used to perform these protein measurements is known to be highly non-quantitative, which may mask subtle differences in expression. Third, beyond gene expression, p50 expression is also subject to a variety of post-transcriptional regulatory mechanisms such as precursor cleavage that might affect the final level of p50 protein, while p65 is not generated from a precursor protein [34]. NF-κB has been shown to be activated by *H. pylori* [35], a known GC carcinogen, and aberrant NF-κB signaling has also been implicated in multiple inflammation-linked cancers such as GC [36]. NF-κB has been suggested to be constitutively activated in primary gastric cancers in a few studies [7]. Targeted NF-κB-inhibitors are currently being actively developed in many anticancer drug development programs and a subset of GC patients (i.e. those with elevated NF-κB activity) may represent a suitable subclass for evaluating the efficacy of these compounds.

The *in silico* method used in our study is conceptually similar to the work of Bild et al, which used a binary regression model to classify tumors based on the predicted activity of five oncogenic pathways [9]. Unlike binary regression, our approach, which makes use of a rank-based connectivity metric [37], requires no elaborate training process on each pathway signature and also does not require the availability of raw expression data, facilitating the use of the many publicly available pathway signatures in the literature [27]. However, the gene expression-based approach does have limitations. First, because our pathway predictions are based on gene expression rather than proteins, such predictions are admittedly molecular surrogates of true pathway signaling activity. Second, we are currently limited to analyzing known oncogenic pathways previously identified in the literature. Third, although we were able to use pathway signatures from very different tissue contexts to predict pathway activation status, an examination of the initial proof-of-principle breast cancer examples revealed that the association of ER status to estrogen responsiveness as predicted using the osteosarcoma signature, although significant, was markedly weaker compared to the association of ER status to tamoxifen sensitivity predicted using a signature derived from the same tissue type (i.e. breast). This result implies that there may also exist tissue-specific differences in pathway signatures that may affect prediction accuracy. Fourth, compared to our study which focused on pathways of known biological relevance in GC, it is unclear if this method can be applied to diseases where prior knowledge of involved pathways may not be available. However, it should be noted that a wealth of pathway signatures (>1000) associated with diverse biochemical and signaling pathways already exists in the literature, which can be accessed from public databases such as MSigDB (http://www.broad.mit.edu/gsea/msigdb/genesets.jsp?collection=CGP). Since our approach can be applied to virtually any disease dataset for which gene expression information is available, testing every signature in a high-throughput manner for evidence of pathway deregulation is both conceivable and feasible. In such cases, pathway exhibiting high frequencies of deregulation would then represent candidate pathways involved in the disease in question, which can then be targeted for focused investigation and experimentation. Addressing these issues will form the ground for much future research.

In conclusion, we have shown in this work that pathways signatures can be successfully used to predict the activation status of cellular signaling pathways, even in biological entities as complex as a human GC. One obvious immediate application of such pathway-based taxonomies may relate to the use of targeted therapies. Initial trials assessing the role of targeted therapies in GC have demonstrated only modest results [38]; however, most of these studies have been performed without pre-stratifying patients using molecular or histopathologic criteria. Pathway-based taxonomies may prove very useful in developing personalized treatment regimens for different subgroups of GC, since such oncogenic pathway activation patterns can be readily linked to potential pathway inhibitors and targeted therapies.

## Materials and Methods

### Primary Gastric Cancer Samples

Three cohorts of gastric cancer were profiled: Cohort 1–70 tumors (Peter MacCallum Cancer Centre, Australia), Cohort 2–200 tumors (National Cancer Centre, Singapore), and Cohort 3–31 tumors (Leeds Institute of Molecular Medicine, United Kingdom). All GCs were collected with approvals from the respective institutions, Research Ethics Review Committee, and signed patient informed consent. Histopathological data of all GC cohorts are provided in Table S8, S9, S10. The median follow-up period was 16.89 months for Cohort 1 and 13.47 months for Cohort 2. 43 patients from Cohort 1 and 91 from Cohort 2 were dead at the end of the study period.

### Gastric Cancer Cell Lines

A total of 25 unique GC cell lines were profiled. GC cell lines AGS, Kato III, SNU1, SNU5, SNU16, NCI-N87, and Hs746T were obtained from the American Type Culture Collection and AZ521, Ist1, TMK1, MKN1, MKN7, MKN28, MKN45, MKN74, Fu97, and IM95 cells were obtained from the Japanese Collection of Research Bioresources/Japan Health Science Research Resource Bank and cultured as recommended. SCH cells were a gift from Yoshiaki Ito (Institute of Molecular and Cell Biology, Singapore) and were grown in RPMI media. YCC1, YCC3, YCC6, YCC7, YCC10, YCC11, and YCC16 cells were a gift from Sun-Young Rha (Yonsei Cancer Center, South Korea) and were grown in MEM supplemented with 10% fetal bovine serum (FBS), 100 units/mL penicillin, 100 units/mL streptomycin, and 2 mmol/L L-glutamine (Invitrogen).

### RNA Extraction and Gene Expression Profiling

Total RNA was extracted from cell lines and primary tumors using Qiagen RNA extraction reagents (Qiagen) according to the instructions of the manufacturer. Cell line and primary tumor mRNAs from Cohort 1 and Cohort 2 were hybridized to Affymetrix Human Genome U133 plus Genechips (HG-U133 Plus 2.0, Affymetrix), while primary tumor mRNAs from Cohort 3 were profiled using U133A Genechips (HG-U133A, Affymetrix). All protocols were performed according to the instructions of the manufacturer. Raw data obtained after chip-scanning was further processed using the MAS5 algorithm (Affymetrix) available in the Bioconductor package *simpleaffy*. The microarray data sets are available at http://www.ncbi.nlm.nih.gov/projects/geo/ (Accession: GSE15460).

### Signatures of Pathway Activation

All signatures used in this study were previously generated [9], [19], [20], [22]–[24], [39]–[49], and obtained from either the MSigDB database [27] (http://www.broad.mit.edu/gsea/msigdb/genesets.jsp?collection=CGP) or original references [9],[39]. Detailed descriptions of the signatures and their sources are available in Table S11 and Table S12. Each signature is represented by a geneset, termed a query signature (QS). Depending on the signature, a QS may consist of only up- (or down-)regulated genes, i.e. genes up- (or down-)regulated during the activation of the pathway. It may also consist of both up- and down-regulated genes. Our approach is capable of handling all of the aforementioned types of QS. QSs were mapped to the probeset domain of the cancer profiles (HG-U133A or HG-U133 Plus 2.0) before computing the pathway activation scores for the cancer profiles. Mapping of QSs were performed using the probe mapping (‘.chip’) files available from ftp://gseaftp.broad.mit.edu/pub/gsea/annotations [27].

For each pathway, we used whenever possible multiple signatures from independent studies, to minimize the possibility of laboratory-specific effects. For further analyses (e.g. survival comparisons), we used the mean of activation scores across independent signatures belonging to the same pathway or group of pathways in order to determine the final activation status of the pathway or group of pathways.

### Mapping Pathway Prediction Signatures in Breast Cancers

Two pathway activation signatures [19],[20] (Table S11) related to the estrogen signaling pathway were analyzed. The breast cancer cell line dataset [18] was obtained from http://www.ebi.ac.uk/microarray-as/ae/download/E-TABM-157.raw.zip. Activation scores for breast cancer cell lines were computed by comparing each individual line against the median profile of the collection of 51 breast cancer cell lines. P-values for the validation of our predictions against ER status were computed using Pearson's chi-square test, under the null hypothesis that the pathway predictor delivers comparable performance to a random predictor.

### Mapping Pathway Prediction Signatures in Gastric Cancers

20 signatures [9], [19], [20], [22]–[24], [39]–[49] (Table S12) representing the activation of 11 pathways related to gastric carcinogenesis were analyzed. Activation scores for primary GCs were computed by comparing each individual GC against the median profile of the patient cohort being analyzed. For the analysis of GC cell lines, final activation scores were obtained by computing the mean activation scores across the seven reference profiles (Table S4). Unsupervised hierarchical clustering (average linkage with centered Pearson correlation metric) was applied to establish patterns of co-activation between different pathways using BRB-ArrayTools.

### Pathway Activation Scores

Pathway activation scores were computed using two inputs: 1) cancer profiles, comprising lists of probesets sorted by differential gene expression between individual cancer gene expression profiles and a reference profile (see Text S1), where *n* is defined as the total number of probesets in each cancer profile *i*, and 2) a query signature QS (pathway activation signature). Probesets representing either up- (or down-) regulated genes in the QS are defined as ‘tags’, and *t* the number of tags in the up- (or down-) regulated portion of the QS. Raw enrichment scores 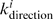 were computed using a Kolmogorov-Smirnov metric previously described in [37]. Here, ‘direction’ in 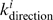 may be considered as ‘up’ or ‘down’, depending on whether the set of tags in question represents the up-regulated (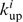) or the down-regulated (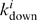) portion of the QS. For a cancer profile *i* and a set of *t* QS tags, the position of tag *j* in the cancer profile *i* is defined as *V*(*j*), forming the vector **V**.
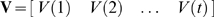
(1)The elements of **V** are then sorted in ascending order of *V*(*j*) such that 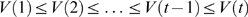. In this manner, the tags indexed by *j* are ordered based on their position in the cancer profile (e.g. tag 1 is the probeset with the highest rank in the cancer profile among all *t* tags in the up- (or down-) regulated portion of the QS). Using the sorted elements of **V**, two parameters are computed:
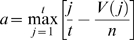
(2)

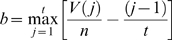
(3)If 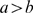, 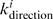 is set to *a*. Otherwise, (if 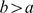), 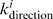 is set to −*b*.

To compute the pathway activation score 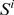, if 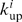 and 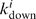 have the same signs then 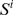 for cancer profile *i* is set to zero. Otherwise, the raw activation score 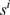
is obtained.
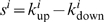
(4)The maximum and minimum of 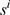 across all cancer profiles in the cohort are defined as *p* and *q*, respectively. The activation score 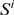 is the normalized form of 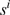, where
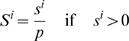
(5)and
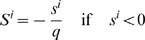
(6)In cases where more than one profile exists for a sample, the final activation score represents the mean activation score across the replicate profiles.

#### Reference profiles

For primary gastric tumor and breast cancer profiles, activation scores were computed using the median profile of the cohort as the reference profile. The median profile was obtained by computing the median of expression values across all members of the cohort. For GCCL profiles, we used seven distinct reference profiles: the median GC cell line profile, a normal skin fibroblast profile, and five normal stomach profiles (Table S4). Besides the median GCCL profile, the other reference profiles were obtained from different cohorts (i.e. different expression datasets). Details regarding the seven reference profiles are available in Table S4 and Text S1. Final activation scores for the GCCLs were obtained by computing the mean activation scores across the seven reference profiles.

### Cell Proliferation Assay

Cell proliferation assays were performed on 22 lines (except SNU1, SNU5, and SNU16) using a CellTiter96 Aqueous Nonradioactive Cell Proliferation Assay kit (Promega) following the manufacturer's instructions. Briefly, cell lines were plated at concentrations of 1×10^3^ to 5×10^3^ cells per well in 96-well plates. Growth rates, representing proliferative activity, were analyzed after 48 hours.

### Western Blotting Assays and Immunocytochemistry

Western blotting was performed as previously described [50] using the following antibodies and dilutions: 1∶500 β-catenin (catalogue number 06-734, Upstate), 1∶500 TCF7L2 (05-511, Upstate), 1∶1,000 β-actin (sc-8432, Santa Cruz), 1∶500 p65 (sc-372, Santa Cruz), 1∶500 p50 (sc-1191, Santa Cruz), and 1∶1,000 GAPDH (ab9483, Abcam). Processing of cell line TMAs (tissue microarrays), blocking, and antigen retrieval was performed as previously described [51]. p65 antibodies were incubated at a dilution of 1∶50 for 2 hours at 37°C. Signal detection was performed using the REAL system (DAKO) at 37°C for 30 minutes, using the DAB chromogen (1∶50 dilution), and Mayer's haematoxylin counterstain. The slides were scored by an experienced histopathologist (H.G.) and the percentage of positive nuclei, percentage of cells with cytoplasmic staining, and staining intensity were assessed.

### Luciferase Reporter Assays

TOPFLASH assays for validation of Wnt/β-catenin activation were performed as previously described [50]. For validation of NF-κB activation, MKN1, MKN7, Hs746T, AGS, and SCH cells were transfected with a pNFκB-Luc reporter (Clontech, Cat. No. 631904) using FuGENE 6 Transfection Reagent (Roche) in 96-well plates. pNFκB-Luc contains the *Photinus pyralis* luciferase gene and multiple copies of the NF-κB consensus sequence fused to a TATA-like promoter region from the Herpes simplex virus thymidine kinase promoter. The same cells were also transfected with pGL4.73[hRluc/SV40] vector (Promega) as a normalization control. Cells were collected 48 hours after transfection and luciferase activity was measured using a dual-luciferase reporter assay system (Promega). All experiments were repeated three independent times.

### Statistical Methods

Kaplan-Meier analysis (SPSS, Chicago) was used for survival comparisons of patient cohorts where clinical follow-up and mortality information were available. P-values representing the significance of the differences in survival outcome (metric: overall survival) were calculated using the Log Rank (Mantel-Cox) test, with p-values of <0.05 being considered significant. Cox regression models were used for computing hazard ratios and implementing multivariate analyses including combined status of two pathways and overall tumor stage (TNM classification: 1–4) as variables. Patients from Cohorts 1 and 2 analyzed in survival comparisons exhibit a significant relationship between overall survival and overall tumor stage, suggesting that patient selection is likely non-biased (data not shown). P-values denoting the significance of a correlation coefficient R between two N-element vectors were estimated from the Student t-distribution, against the null hypothesis that the observed value of t = R/√[(1−R^2^)/(N−2)] comes from a population in which the true correlation coefficient is zero. Unless otherwise specified, all other p-values (used in comparisons of two groups) were computed using Student's t-test. All p-values are two-tailed. Gene Set Enrichment Analysis (GSEA) was performed as described in Subramanian et al. [27].

## Supporting Information

Figure S1 Predictions using pathway signatures or key pathway genes. (A–C) Cell proliferation predictions. Experimentally determined proliferative capacities of GC cell lines were compared against predictions by (A) Myc gene expression, (B) E2F1 gene expression, and (C) the mean activation score from proliferation/stem cell pathway signatures. Both Myc and E2F1 are key proliferation pathway genes. The y-axis represents true proliferative capacity, and the x-axis represents the predictions. While there is no significant correlation using E2F1 or Myc as predictors (p>0.05 in both cases) (A and B), the mean proliferation/stem cell signature score is significantly correlated with proliferative capacity (p = 0.0278) (C) and Figure 4A in Main Text. (D–F) Wnt pathway predictions. Wnt pathway activity was determined in GC cell lines using a TCF4 (aka TCF7L2)-luciferase reporter assay (see Materials and Methods), and compared against predictions by (D) TCF4 gene expression, (E) β-catenin gene expression, and (F) the mean activation score from Wnt/β-catenin signatures. Both TCF4 and β-catenin are key Wnt pathway genes. The y-axis represents true Wnt activity, while the x-axis represents the predictions. While there is no significant correlation using TCF4 or β-catenin as predictors (p>0.05 in both cases) (D and E), the mean Wnt/β-catenin signature activation score is significantly correlated with Wnt activity (p = 0.0380) (F).
(0.05 MB DOC)

Figure S2 Using multiple references to obtain high-confidence prediction of the activation status of the NF-κB pathway. GCCLs ranked top (or bottom) five via at least one of the two NF-κB signatures and at least seven times across all references and signatures were chosen as GCCLs in which the NF-κB pathway is called as activated (‘NF-κB/on’) (or nonactive (‘NF-κB/off’)). Only GCCLs consistently predicted as NF-κB-activated (or NF-κB-nonactive) were chosen for further dry lab and wet bench analyses.
(0.10 MB DOC)

Figure S3 NF-κB immunocytochemistry in gastric cancer cell lines. (A) MKN1 cells show strong cytoplasmic staining in most cells, and nuclear expression of NF-κB in a subset of cells (blue arrow). (B) Hs746T cells show strong cytoplasmic staining in all cells. No nuclear expression of NF-κB. (C) AGS cells show weak cytoplasmic staining in all cells. No nuclear expression of NF-κB. (D) SCH cells show weak cytoplasmic staining in all cells. No nuclear expression of NF-κB. (Chromogen used: DAB (brown), Mayer's haemalaun counterstain (blue), Scale bar = 30 µm)
(7.22 MB DOC)

Figure S4 p50 and p65 gene expression in GCCLs. Gene expression values for p50 and p65 (log10 transformed) across 11 GCCLs were compared. p50 values are plotted as yellow columns, while p65 values are in black. The y-axis represents expression values, while individual GCCLs are on the x-axis sorted by expression level. The range in p50 gene expression is 0.54 or 3.49-fold (10^0.54^ = 3.49), while the range in p65 expression is 1.04 or 10.94-fold. Thus, there is a 3.13× greater degree of range in p65 expression than in p50 expression.
(0.03 MB DOC)

Table S1 Prediction accuracies of estrogen signaling related signatures. (A) Predictions using the breast-derived ‘tamoxifen sensitivity’ signature. (B) Predictions using the osteosarcoma-derived ‘estrogen response’ signature.
(0.03 MB DOC)

Table S2 Membership of the signatures, determined using unsupervised hierarchical clustering in each of the three GC cohorts.
(0.04 MB DOC)

Table S3 Pathway activation frequencies in GC.
(0.03 MB DOC)

Table S4 Reference profiles for gastric cancer cell lines (GCCLs). (A) Descriptions of reference profiles. (B) Pearson correlation values between activation scores from seven different reference profiles used to generate GCCL activation profiles.
(0.04 MB DOC)

Table S5 Summary of results from IHC assay.
(0.03 MB DOC)

Table S6 Multivariate analysis for tumor stage (TNM classification) and combined activation levels of proliferation/stem cell and NF-κB pathways in primary tumors.
(0.04 MB DOC)

Table S7 Multivariate analysis for tumor stage (TNM classification) and combined activation levels of proliferation/stem cell and Wnt/β-catenin pathways in primary tumors.
(0.04 MB DOC)

Table S8 Histopathological data for Cohort 1 of 70 tumors from Australia.
(0.14 MB DOC)

Table S9 Histopathological data for Cohort 2 of 200 tumors from Singapore.
(0.36 MB DOC)

Table S10 Histopathological data for Cohort 3 of 31 tumors from the United Kingdom.
(0.07 MB DOC)

Table S11 Signatures associated with perturbed estrogen signaling.
(0.03 MB DOC)

Table S12 Signatures associated with 11 oncogenic pathways implicated in gastric carcinogenesis.
(0.07 MB DOC)

Text S1 Supplementary methods.
(0.05 MB DOC)
