# Supplementary material for: Oncogenic Pathway Combinations Predict Clinical Prognosis in Gastric Cancer
Source: PLoS Genet. 2009 Oct 2;5(10):e1000676. doi: 10.1371/journal.pgen.1000676 (PMC2748685; doi:10.1371/journal.pgen.1000676)
Supplement: Figure S1 — Predictions using pathway signatures or key pathway genes. (A–C) Cell proliferation predictions. Experimentally determined proliferative capacities of GC cell lines were compared against predictions by (A) Myc gene expression, (B) E2F1 gene expression, and (C) the mean activation score from proliferation/stem cell pathway signatures. Both Myc and E2F1 are key proliferation pathway genes. The y-axis represents true proliferative capacity, and the x-axis represents the predictions. While there is no significant correlation using E2F1 or Myc as predictors (p>0.05 in both cases) (A and B), the mean proliferation/stem cell signature score is significantly correlated with proliferative capacity (p = 0.0278) (C) and Figure 4A in Main Text. (D–F) Wnt pathway predictions. Wnt pathway activity was determined in GC cell lines using a TCF4 (aka TCF7L2)-luciferase reporter assay (see Materials and Methods), and compared against predictions by (D) TCF4 gene expression, (E) β-catenin gene expression, and (F) the mean activation score from Wnt/β-catenin signatures. Both TCF4 and β-catenin are key Wnt pathway genes. The y-axis represents true Wnt activity, while the x-axis represents the predictions. While there is no significant correlation using TCF4 or β-catenin as predictors (p>0.05 in both cases) (D and E), the mean Wnt/β-catenin signature activation score is significantly correlated with Wnt activity (p = 0.0380) (F). (0.05 MB DOC) [file pgen.1000676.s001.doc]

Figure S1. Predictions using pathway signatures or key pathway genes.

A-C) Cell proliferation predictions. Experimentally determined proliferative capacities of GC cell lines were compared against predictions by A) Myc gene expression, B) E2F1 gene expression, and C) the mean activation score from proliferation/stem cell pathway signatures. Both Myc and E2F1 are key proliferation pathway genes. The y-axis represents true proliferative capacity, and the x-axis represents the predictions. While there is no significant correlation using E2F1 or Myc as predictors (p>0.05 in both cases) (A and B), the mean proliferation/stem cell signature score is significantly correlated with proliferative capacity (p=0.0278) (C and Figure 4A in Main Text)

D-F) Wnt pathway predictions. Wnt pathway activity was determined in GC cell lines using a TCF4 (aka TCF7L2)-luciferase reporter assay (see Methods), and compared against predictions by D) TCF4 gene expression, E) β-catenin gene expression, and F) the mean activation score from Wnt/β-catenin signatures. Both TCF4 and β-catenin are key Wnt pathway genes. The y-axis represents true Wnt activity, while the x-axis represents the predictions. While there is no significant correlation using TCF4 or β-catenin as predictors (p>0.05 in both cases) (D and E), the mean Wnt/β-catenin signature activation score is significantly correlated with Wnt activity (p=0.0380) (F).
