# Supplementary material for: Oncogenic Pathway Combinations Predict Clinical Prognosis in Gastric Cancer
Source: PLoS Genet. 2009 Oct 2;5(10):e1000676. doi: 10.1371/journal.pgen.1000676 (PMC2748685; doi:10.1371/journal.pgen.1000676)
Supplement: Figure S2 — Using multiple references to obtain high-confidence prediction of the activation status of the NF-κB pathway. GCCLs ranked top (or bottom) five via at least one of the two NF-κB signatures and at least seven times across all references and signatures were chosen as GCCLs in which the NF-κB pathway is called as activated (‘NF-κB/on’) (or nonactive (‘NF-κB/off’)). Only GCCLs consistently predicted as NF-κB-activated (or NF-κB-nonactive) were chosen for further dry lab and wet bench analyses. (0.10 MB DOC) [file pgen.1000676.s002.doc]

Figure S2. Using multiple references to obtain high-confidence prediction of the activation status of the NF-κB pathway.

GCCLs ranked top (or bottom) five via at least one of the two NF-κB signatures and at least seven times across all references and signatures were chosen as GCCLs in which the NF-κB pathway is called as activated (‘NF-κB/on’) (or nonactive (‘NF-κB/off’)). Only GCCLs consistently predicted as NF-κB-activated (or NF-κB-nonactive) were chosen for further dry lab and wet bench analyses.
