# Supplementary material for: Oncogenic Pathway Combinations Predict Clinical Prognosis in Gastric Cancer
Source: PLoS Genet. 2009 Oct 2;5(10):e1000676. doi: 10.1371/journal.pgen.1000676 (PMC2748685; doi:10.1371/journal.pgen.1000676)
Supplement: Figure S3 — NF-κB immunocytochemistry in gastric cancer cell lines. (A) MKN1 cells show strong cytoplasmic staining in most cells, and nuclear expression of NF-κB in a subset of cells (blue arrow). (B) Hs746T cells show strong cytoplasmic staining in all cells. No nuclear expression of NF-κB. (C) AGS cells show weak cytoplasmic staining in all cells. No nuclear expression of NF-κB. (D) SCH cells show weak cytoplasmic staining in all cells. No nuclear expression of NF-κB. (Chromogen used: DAB (brown), Mayer's haemalaun counterstain (blue), Scale bar = 30 µm) (7.22 MB DOC) [file pgen.1000676.s003.doc]

Figure S3. NF-κB immunocytochemistry in gastric cancer cell lines.

A) MKN1 cells show strong cytoplasmic staining in most cells, and nuclear expression of NF-κB in a subset of cells (blue arrow)

B) Hs746T cells show strong cytoplasmic staining in all cells. No nuclear expression of NF-κB.

C) AGS cells show weak cytoplasmic staining in all cells. No nuclear expression of NF-κB.

D) SCH cells show weak cytoplasmic staining in all cells. No nuclear expression of NF-κB.

(Chromogen used: DAB (brown), Mayer’s haemalaun counterstain (blue), Scale bar = 30µm)
