# Supplementary material for: Oncogenic Pathway Combinations Predict Clinical Prognosis in Gastric Cancer
Source: PLoS Genet. 2009 Oct 2;5(10):e1000676. doi: 10.1371/journal.pgen.1000676 (PMC2748685; doi:10.1371/journal.pgen.1000676)
Supplement: Figure S4 — p50 and p65 gene expression in GCCLs. Gene expression values for p50 and p65 (log10 transformed) across 11 GCCLs were compared. p50 values are plotted as yellow columns, while p65 values are in black. The y-axis represents expression values, while individual GCCLs are on the x-axis sorted by expression level. The range in p50 gene expression is 0.54 or 3.49-fold (100.54 = 3.49), while the range in p65 expression is 1.04 or 10.94-fold. Thus, there is a 3.13× greater degree of range in p65 expression than in p50 expression. (0.03 MB DOC) [file pgen.1000676.s004.doc]

Figure S4. p50 and p65 gene expression in GCCLs.

Gene expression values for p50 and p65 (log10 transformed) across 11 GCCLs were compared. p50 values are plotted as yellow columns, while p65 values are in black. The y-axis represents expression values, while individual GCCLs are on the x-axis sorted by expression level. The range in p50 gene expression is 0.54 or 3.49-fold (10^0.54^= 3.49), while the range in p65 expression is 1.04 or 10.94-fold. Thus, there is a 3.13x greater degree of range in p65 expression than in p50 expression.
