# Supplementary material for: Oncogenic Pathway Combinations Predict Clinical Prognosis in Gastric Cancer
Source: PLoS Genet. 2009 Oct 2;5(10):e1000676. doi: 10.1371/journal.pgen.1000676 (PMC2748685; doi:10.1371/journal.pgen.1000676)
Supplement: Table S1 — Prediction accuracies of estrogen signaling related signatures. (A) Predictions using the breast-derived ‘tamoxifen sensitivity’ signature. (B) Predictions using the osteosarcoma-derived ‘estrogen response’ signature. (0.03 MB DOC) [file pgen.1000676.s005.doc]

Table S1. Prediction accuracies of estrogen signaling related signatures

A) Predictions using the breast-derived ‘tamoxifen sensitivity’ signature

| **ER status:**  **Predicted tamoxifen sensitivity** | **ER-positive** | **ER-negative** |
| --- | --- | --- |
| Sensitive (score > 0) | 19 | 8 |
| Resistant (score ≤ 0) | 0 | 24 |

B) Predictions using the osteosarcoma-derived ‘estrogen response’ signature

| **ER status:**  **Predicted estrogen responsiveness** | **ER-positive** | **ER-negative** |
| --- | --- | --- |
| Responsive (score > 0) | 18 | 18 |
| Non-responsive (score ≤ 0) | 1 | 14 |

ER status (positive or negative) was based on *ESR1* (estrogen receptor) expression in a panel of 51 breast cancer cell lines [1]. The accuracy of the tamoxifen sensitivity signature is 84.3% (p=2.12×10^-7^, Sensitivity 100%, Specificity 75%), while the accuracy of the estrogen response signature is 62.7% (p=0.0035, Sensitivity 94.7%, Specificity 43.8%).

**Reference**

1. Neve RM, Chin K, Fridlyand J, Yeh J, Baehner FL, et al. (2006) A collection of breast cancer cell lines for the study of functionally distinct cancer subtypes. Cancer Cell 10: 515-527.
