# Supplementary material for: Oncogenic Pathway Combinations Predict Clinical Prognosis in Gastric Cancer
Source: PLoS Genet. 2009 Oct 2;5(10):e1000676. doi: 10.1371/journal.pgen.1000676 (PMC2748685; doi:10.1371/journal.pgen.1000676)
Supplement: Table S2 — Membership of the signatures, determined using unsupervised hierarchical clustering in each of the three GC cohorts. (0.04 MB DOC) [file pgen.1000676.s006.doc]

Table S2. Membership of the signatures, determined using unsupervised hierarchical clustering in each of the three GC cohorts.

| **Signature** | **Clustering membership in Australia cohort** | **Clustering membership in Singapore cohort** | **Clustering membership in UK cohort** |
| --- | --- | --- | --- |
| E2F (osteosarcoma) | 1 | 1 | 1 |
| E2F (rat) | 1 | 1 | 1 |
| p21-repressed | 1 | 1 | 1 |
| Stem cell (ESC) | 1 | 1 | 1 |
| Stem cell (ESC) | 1 | 1 | 1 |
| Stem cell (NSC) | 1 | 1 | 1 |
| MYC (umbilical) | 1 | 1 | 1 |
| MYC (breast) | 1 | 1 | 2 |
| p53 (lung) | 2 | Outlier | 2 |
| NF-κB (skin) | 1 | 1 | 1 |
| NF-κB (cervix) | 1 | 2 | 1 |
| Wnt | 1 | 1 | 1 |
| p53 (mouse) | 2 | 2 | 1 |
| BRCA1 (kidney) | 2 | Outlier | 1 |
| BRCA1 (prostate) | 2 | 2 | 2 |
| RAS | 2 | 2 | 2 |
| β-catenin | 2 | 2 | 2 |
| HDAC inhibition (BUT) | 2 | Outlier | 2 |
| HDAC inhibition (TSA) | 2 | 2 | 2 |
| SRC | 2 | 2 | 2 |

“1” indicates that a signature is in the cluster containing the E2F, p21-repression, and stem cell signatures; “2” indicates that a signature is in the other cluster. “Outlier” indicates that the signature belongs to neither cluster and is isolated from the main tree containing the two branches (“1” and “2”).

The p-values were computed using Pearson's chi-square (χ2) test. The p-values for comparisons of the Australia cohort to the Singapore and UK cohorts, p=0.00038 and p=0.00051, respectively, enable the rejection of the null hypotheses that cluster membership seen in a tumor cohort (Singapore or UK) is random, with no correlation to the clustering in the Australia cohort. The p-value for the comparison between the Singapore and UK cohorts is p= 0.0075, enabling the rejection of the null hypothesis that cluster membership seen in the UK cohort is random, with no correlation to the clustering in the Singapore cohort. These p-values indicate that the similarity of overall patterns of pathway activation observed across all three GC cohorts is significant.
