# Supplementary material for: Oncogenic Pathway Combinations Predict Clinical Prognosis in Gastric Cancer
Source: PLoS Genet. 2009 Oct 2;5(10):e1000676. doi: 10.1371/journal.pgen.1000676 (PMC2748685; doi:10.1371/journal.pgen.1000676)
Supplement: Table S3 — Pathway activation frequencies in GC. (0.03 MB DOC) [file pgen.1000676.s007.doc]

Table S3. Pathway activation frequencies in GC.

| **Pathway** | **% in Cohort 1** | **% in Cohort 2** | **% in Cohort 3** | **% in all cohorts** |
| --- | --- | --- | --- | --- |
| MYC | 40.00 | 45.71 | 38.71 | 41.20 |
| Stem cell | 43.00 | 40.00 | 38.71 | 41.86 |
| E2F | 45.00 | 40.00 | 45.16 | 43.85 |
| p21-repression | 42.00 | 47.14 | 48.39 | 43.85 |
| NF-κB | 39.50 | 41.43 | 35.48 | 39.53 |
| Wnt/β-catenin | 47.00 | 42.86 | 48.39 | 46.18 |
| p53 | 46.50 | 48.57 | 48.39 | 47.18 |
| SRC | 38.00 | 41.43 | 25.81 | 37.54 |
| RAS | 32.00 | 30.00 | 32.26 | 31.56 |
| BRCA1 | 23.50 | 27.14 | 22.58 | 24.25 |
| HDACI | 31.50 | 37.14 | 29.03 | 32.56 |

Tumors with positive mean activation scores (>0) across signatures from the pathway were considered to show pathway activation.
