# Supplementary material for: Oncogenic Pathway Combinations Predict Clinical Prognosis in Gastric Cancer
Source: PLoS Genet. 2009 Oct 2;5(10):e1000676. doi: 10.1371/journal.pgen.1000676 (PMC2748685; doi:10.1371/journal.pgen.1000676)
Supplement: Table S4 — Reference profiles for gastric cancer cell lines (GCCLs). (A) Descriptions of reference profiles. (B) Pearson correlation values between activation scores from seven different reference profiles used to generate GCCL activation profiles. (0.04 MB DOC) [file pgen.1000676.s008.doc]

Table S4. Reference profiles for gastric cancer cell lines (GCCLs).

A) Descriptions of reference profiles.

| **No.** | **Reference name** | **Details** | **#Replicates or profiles** | **GEO Accession #** |
| --- | --- | --- | --- | --- |
| 1 | Median GCCL | Median of GC cell lines | 1 | GSE15455 |
| 2 | CRL2072 | Normal skin fibroblasts, 3 replicates | 3 | GSE15455 |
| 3 | STN_CARDIAC | Stomach cardiac, 2 male and 1 female profiles | 3 | GSE7307 |
| 4 | STN_PYLORIC | Stomach pyloric, 3 male and 1 female profiles | 4 | GSE7307 |
| 5 | STN_FUNDUS | Stomach fundus, 3 male and 1 female profiles | 4 | GSE7307 |
| 6 | STN_BD | Normal stomach, 1 profile from M/F mix | 1 | GSE7307 |
| 7 | STN_GSE7307 | Combination of 3-6 | 12 | GSE7307 |

B) Pearson correlation values between activation scores from seven different reference profiles used to generate GCCL activation profiles.

| **Reference** | Median GCCL | CRL2072 | STN_CARDIAC | STN_PYLORIC | STN_FUNDUS | STN_BD | STN_GSE7307 |
| --- | --- | --- | --- | --- | --- | --- | --- |
| Median GCCL | 1.0000 | 0.2347 | 0.2159 | 0.1993 | 0.1957 | 0.1811 | 0.1956 |
| CRL2072 |  | 1.0000 | 0.6080 | 0.6915 | 0.6786 | 0.6663 | 0.6890 |
| STN_CARDIAC |  |  | 1.0000 | 0.8870 | 0.8773 | 0.9056 | 0.9142 |
| STN_PYLORIC |  |  |  | 1.0000 | 0.9140 | 0.9383 | 0.9518 |
| STN_FUNDUS |  |  |  |  | 1.0000 | 0.8857 | 0.9450 |
| STN_BD |  |  |  |  |  | 1.0000 | 0.9287 |
| STN_GSE7307 |  |  |  |  |  |  | 1.0000 |

All pairwise correlations are positive and all associated p-values are < 1×10^−4^, indicating that any two different reference profiles are more likely to be in concordance when predicting pathway activation than not. The weakest correlation occurred between activation scores computed using the GCCL median profile and those computed using the STN_BD profile (non-malignant stomach profile), with a correlation of 0.1811 (p= 2.32568×10^−5^).
