# Supplementary material for: Oncogenic Pathway Combinations Predict Clinical Prognosis in Gastric Cancer
Source: PLoS Genet. 2009 Oct 2;5(10):e1000676. doi: 10.1371/journal.pgen.1000676 (PMC2748685; doi:10.1371/journal.pgen.1000676)
Supplement: Table S5 — Summary of results from IHC assay. (0.03 MB DOC) [file pgen.1000676.s009.doc]

Table S5. Summary of results from IHC assay.

| **GCCL** | **Predicted NF-κB activation status** | **Nuclear staining (% cells)** | **Cytoplasm staining (% cells)** | **Strength of staining** |
| --- | --- | --- | --- | --- |
| MKN1 | On | 20 | 100 | strong |
| SNU5 | On | 0 | 85 | strong |
| YCC16 | On | 0 | 100 | *weak* |
| Hs746T | On | 0 | 100 | strong |
| MKN7 | On | 0 | 100 | strong |
| YCC6 | On | 0 | 100 | strong |
| SCH | Off | 0 | 100 | *weak* |
| SNU16 | Off | 0 | 90 | moderate |
| AGS | Off | 0 | 60 | *weak* |
| SNU1 | Off | 0 | 100 | strong |
| TMK1 | Off | 0 | 100 | strong |

Note: GCCLs have been arranged based on predicted NF-κB activation, from highest (MKN1) to lowest (TMK1).
