# Supplementary material for: Oncogenic Pathway Combinations Predict Clinical Prognosis in Gastric Cancer
Source: PLoS Genet. 2009 Oct 2;5(10):e1000676. doi: 10.1371/journal.pgen.1000676 (PMC2748685; doi:10.1371/journal.pgen.1000676)
Supplement: Table S6 — Multivariate analysis for tumor stage (TNM classification) and combined activation levels of proliferation/stem cell and NF-κB pathways in primary tumors. (0.04 MB DOC) [file pgen.1000676.s010.doc]

Table S6. Multivariate analysis for tumor stage (TNM classification) and combined activation levels of proliferation/stem cell and NF-κB pathways in primary tumors.

| **Data Set** | **p-value** | **Hazard ratio** | **(95% CI)** | |
| --- | --- | --- | --- | --- |
| **Cohort 1** |  |  | Lower | Upper |
| Stage 1A (ref) | .009 |  |  |  |
| 1B | .185 | .125 | .006 | 2.712 |
| 2 | .084 | .125 | .012 | 1.319 |
| 3A | .137 | .174 | .017 | 1.746 |
| 3B | .586 | .518 | .049 | 5.525 |
| 4 | .920 | 1.133 | .099 | 13.021 |
| High in both proliferation/stem cell and NF-κB pathways (ref) | **.003** |  |  |  |
| High in only either pathway | .002 | .177 | .060 | .522 |
| Low in both proliferation/stem cell and NF-κB pathways | .001 | .167 | .057 | .488 |
|  |  |  |  |  |
| **Cohort 2** |  |  |  |  |
| Stage 1A (ref) | .001 |  |  |  |
| 1B | .802 | 1.200 | .288 | 4.996 |
| 2 | .482 | 1.599 | .431 | 5.927 |
| 3A | .153 | 2.499 | .712 | 8.777 |
| 3B | .031 | 4.246 | 1.140 | 15.823 |
| 4 | .009 | 5.226 | 1.520 | 17.975 |
| High in both proliferation/stem cell and NF-κB pathways (ref) | **.048** |  |  |  |
| High in only either pathway | .937 | .972 | .477 | 1.979 |
| Low in both proliferation/stem cell and NF-κB pathways | .092 | .548 | .272 | 1.103 |

Note: Significant p-values are in bold.
