# Supplementary material for: Oncogenic Pathway Combinations Predict Clinical Prognosis in Gastric Cancer
Source: PLoS Genet. 2009 Oct 2;5(10):e1000676. doi: 10.1371/journal.pgen.1000676 (PMC2748685; doi:10.1371/journal.pgen.1000676)
Supplement: Table S7 — Multivariate analysis for tumor stage (TNM classification) and combined activation levels of proliferation/stem cell and Wnt/β-catenin pathways in primary tumors. (0.04 MB DOC) [file pgen.1000676.s011.doc]

Table S7. Multivariate analysis for tumor stage (TNM classification) and combined activation levels of proliferation/stem cell and Wnt/β-catenin pathways in primary tumors.

| **Data Set** | **p-value** | **Hazard ratio** | **(95% CI)** | |
| --- | --- | --- | --- | --- |
| **Cohort 1** |  |  | Lower | Upper |
| Stage 1A (ref) | .010 |  |  |  |
| 1B | .242 | .158 | .007 | 3.483 |
| 2 | .105 | .143 | .014 | 1.503 |
| 3A | .189 | .215 | .022 | 2.128 |
| 3B | .742 | .669 | .061 | 7.287 |
| 4 | .994 | 1.008 | .100 | 10.185 |
| High in both proliferation/stem cell and Wnt/β-catenin pathways (ref) | **<.001** |  |  |  |
| High in only either pathway | .000 | .107 | .032 | .351 |
| Low in both proliferation/stem cell and Wnt/ β-catenin pathways | .000 | .118 | .041 | .337 |
|  |  |  |  |  |
| **Cohort 2** |  |  |  |  |
| Stage 1A (ref) | .003 |  |  |  |
| 1B | .775 | .814 | .198 | 3.341 |
| 2 | .821 | 1.160 | .320 | 4.205 |
| 3A | .346 | 1.804 | .529 | 6.157 |
| 3B | .103 | 2.842 | .810 | 9.970 |
| 4 | .036 | 3.707 | 1.088 | 12.624 |
| High in both proliferation/stem cell and Wnt/ β-catenin pathways (ref) | .058 |  |  |  |
| High in only either pathway | .111 | .622 | .347 | 1.116 |
| Low in both proliferation/stem cell and Wnt/ β-catenin pathways | .017 | .464 | .247 | .872 |

Note: Significant p-values are in bold
