# Supplementary material for: Oncogenic Pathway Combinations Predict Clinical Prognosis in Gastric Cancer
Source: PLoS Genet. 2009 Oct 2;5(10):e1000676. doi: 10.1371/journal.pgen.1000676 (PMC2748685; doi:10.1371/journal.pgen.1000676)
Supplement: Table S8 — Histopathological data for Cohort 1 of 70 tumors from Australia. (0.14 MB DOC) [file pgen.1000676.s012.doc]

Table S8. Histopathological data for Cohort 1 of 70 tumors from Australia

| **Expt number** | **Age** | **Gender** | **Lauren** | **Differentiation** | **Stage** | **Margins** |
| --- | --- | --- | --- | --- | --- | --- |
| GCa0001 | 73 | M | Intestinal | Moderate | 3a | R0 |
| GCa0002 | 70 | M | Intestinal | Moderate | 3a | R0 |
| GCa0006 | 85 | F | Intestinal | Poor | 3b | R0 |
| GCa0008 | 61 | M | Diffuse | Undifferentiated | 3a | R0 |
| GCa0010 | 68 | F | Diffuse | Undifferentiated | 2 | R0 |
| GCa0012 | 83 | F | Diffuse | Poor | 1b | R0 |
| GCa0014 | 64 | F | Intestinal | Well | 2 | R0 |
| GCa0015 | 66 | M | Intestinal | Poor | 4 | R0 |
| GCa0016 | 78 | M | Intestinal | Moderate | 2 | R0 |
| GCa0018 | 65 | M | Intestinal | Moderate | 1a | R0 |
| GCa0019 | 32 | M | Intestinal | Poor | 3b | R0 |
| GCa0020 | 64 | M | Diffuse | Poor | 1b | R0 |
| GCa0021 | 54 | M | Diffuse | Undifferentiated | 3a | R0 |
| GCa0023 | 47 | F | Diffuse | Undifferentiated | 1b | R0 |
| GCa0024 | 55 | M | Intestinal | Well | 3b | R0 |
| GCa0026 | 61 | F | Intestinal | Moderate | 2 | R1 |
| GCa0028 | 70 | F | Intestinal | Moderate | 3a | R0 |
| GCa0029 | 79 | F | Intestinal | Poor | 2 | R0 |
| GCa0031 | 60 | M | Intestinal | Poor | 4 | R0 |
| GCa0032 | 80 | F | Diffuse | Undifferentiated | 1b | R0 |
| GCa0033 | 33 | F | Diffuse | Poor | 3a | R0 |
| GCa0034 | 79 | F | Mixed | Poor | 3b | R0 |
| GCa0036 | 81 | M | Diffuse | Poor | 3a | R0 |
| GCa0037 | 76 | F | Diffuse | Undifferentiated | 3b | R0 |
| GCa0039 | 75 | M | Diffuse | Poor | 3b | R0 |
| GCa0041 | 55 | M | Diffuse | Undifferentiated | 3a | R0 |
| GCa0043 | 57 | M | Intestinal | Moderate | 3a | R0 |
| GCa0045 | 77 | F | Intestinal | Poor | 1b | R0 |
| GCa0046 | 73 | F | Intestinal | Moderate | 2 | R0 |
| GCa0047 | 69 | M | Intestinal | Moderate | 3a | R0 |
| GCa0049 | 74 | M | Intestinal | Moderate | 2 | R0 |
| GCa0050 | 67 | M | Mixed | Poor | 3b | R0 |
| GCa0051 | 56 | M | Mixed | Poor | 4 | R0 |
| GCa0052 | 67 | M | Intestinal | Moderate | 2 | R0 |
| GCa0053 | 77 | F | Intestinal | Moderate | 2 | R0 |
| GCa0055 | 76 | F | Diffuse | Undifferentiated | 2 | R1 |
| GCa0057 | 79 | M | Intestinal | Moderate | 2 | R0 |
| GCa0058 | 73 | M | Diffuse | Undifferentiated | 4 | R0 |
| GCa0060 | 69 | M | Intestinal | Moderate | 2 | R0 |
| GCa0062 | 74 | F | Diffuse | Undifferentiated | 3b | R1 |
| GCa0063 | 75 | F | Diffuse | Undifferentiated | 1b | R0 |
| GCa0065 | 69 | M | Diffuse | Poor | 1b | R0 |
| GCa0068 | 60 | M | Intestinal | Moderate | 3a | R0 |
| GCa0070 | 36 | M | Diffuse | Poor | 3b | R0 |
| GCa0072 | 59 | F | Diffuse | Poor | 1b | R0 |
| GCa0073 | 70 | M | Intestinal | Moderate | 3a | R0 |
| GCa0074 | 62 | M | Diffuse | Poor | 3b | R0 |
| GCa0079 | 57 | M | Intestinal | Poor | 1b | R0 |
| GCa0080 | 74 | M | Intestinal | Moderate | 1a | R0 |
| GCa0082 | 62 | M | Diffuse | Undifferentiated | 4 | R0 |
| GCa0083 | 82 | M | Diffuse | Undifferentiated | 3b | R0 |
| GCa0086 | 67 | F | Intestinal | Moderate | 3a | R0 |
| GCa0087 | 53 | M | Intestinal | Moderate | 1b | R0 |
| GCa0089 | 57 | M | Diffuse | Undifferentiated | 3b | R1 |
| GCa0091 | 83 | M | Intestinal | Moderate | 2 | R0 |
| GCa0093 | 47 | M | Diffuse | Undifferentiated | 3a | R0 |
| GCa0095 | 66 | M | Intestinal | Moderate | 4 | R0 |
| GCa0096 | 50 | M | Diffuse | Undifferentiated | 3b | R0 |
| GCa0097 | 81 | F | Diffuse | Undifferentiated | 3b | R0 |
| GCa0099 | 80 | M | Mixed | Undifferentiated | 3a | R0 |
| GCa0101 | 71 | M | Diffuse | Undifferentiated | 3b | R0 |
| GCa0103 | 71 | M | Mixed | Poor | 3a | R0 |
| GCa0105 | 54 | M | Intestinal | Poor | 1b | R0 |
| GCa0109 | 44 | M | Diffuse | Undifferentiated | 3a | R0 |
| GCa0111 | 50 | F | Diffuse | Poor | 4 | R0 |
| GCa0113 | 51 | M | Intestinal | Poor | 2 | R0 |
| GCa0114 | 65 | M | Intestinal | Poor | 2 | R0 |
| GCa0115 | 85 | M | Diffuse | Poor | . | R0 |
| GCa0116 | 56 | M | Mixed | Poor | 2 | R0 |
| GCa0117 | 49 | M | Intestinal | Moderate | 3a | R0 |
