# Supplementary material for: Oncogenic Pathway Combinations Predict Clinical Prognosis in Gastric Cancer
Source: PLoS Genet. 2009 Oct 2;5(10):e1000676. doi: 10.1371/journal.pgen.1000676 (PMC2748685; doi:10.1371/journal.pgen.1000676)
Supplement: Table S9 — Histopathological data for Cohort 2 of 200 tumors from Singapore. (0.36 MB DOC) [file pgen.1000676.s013.doc]

Table S9. Histopathological data for Cohort 2 of 200 tumors from Singapore

| **TR Number** | **Age** | **Gender** | **Type** | **Lauren** | **Differentiation** | **T stage** | **Ming** | **Nodes** | **Grade** |
| --- | --- | --- | --- | --- | --- | --- | --- | --- | --- |
| 980056 | 33 | F | Adeno |  | Poor | T3 |  | N0 |  |
| 970005 | 68 | M | Adeno | Intestinal | Mod | T3 |  | N0 | G2 |
| 970003 | 38 | F | Adeno |  | Poor | T3 |  | N1 |  |
| 970001 | 51 | F | Adeno |  | Poor | T3 |  | N1 |  |
| 980048 | 48 | F | Adeno |  | Poor | T3 |  | N0 | G3 |
| 980029 | 75 | M | Adeno | Diffuse | Poor | T2 |  | N1 |  |
| 980028 | 58 | M | Adeno |  | Mod | T2 |  | N0 |  |
| 980025 | 80 | M | Adeno |  | Poor | T3 |  | N3 |  |
| 980021 | 59 | M | Adeno |  | Mod | T3 |  | N2 |  |
| 980011 | 74 | M | Adeno |  | Mod | T3 |  | N1 |  |
| 970010 | 72 | M | Adeno |  | Mod | T3 |  | N1 |  |
| 970017 | 57 | F | Adeno |  | Poor | T3 |  | N0 |  |
| 970032 | 55 | F | Adeno |  |  | T1 |  | N0 |  |
| 970024 | 80 | M | Adeno |  | Mod | T3 |  | N0 |  |
| 980035 | 40 | M | Adeno | Intestinal | Mod | T3 |  | N2 |  |
| 980002 | 58 | F | Adeno |  | Poor | T2 |  | N1 |  |
| 980148 | 66 | M | Adeno |  | Poor | T3 |  | N1 |  |
| 980156 | 73 | F | Carcinoid |  | Poor |  |  |  |  |
| 980161 | 66 | F | Adeno |  | Poor | T3 |  | N1 |  |
| 980184 | 74 | F | Adeno |  | Mod | T3 |  | N2 |  |
| 980211 | 63 | M | Adeno | Intestinal | Mod | T3 |  | N2 |  |
| 980252 |  | F | Adeno |  | Poor | T3 |  | N1 |  |
| 980255 | 74 | M | Adeno |  | Mod | T3 |  | N1 |  |
| 980251 | 52 | F | Adeno | Mixed |  | T1 |  | N0 |  |
| 980269 | 68 | M | Adeno | Diffuse | Poor | T3 |  | N3 |  |
| 980305 | 38 | F | Adeno |  | Poor | T3 |  | N3 |  |
| 980307 | 23 | M | Adeno |  | Mod | T3 |  | N2 |  |
| 980097 | 65 | M | Adeno |  |  | T2 |  | N1 |  |
| 980095 | 48 | F | Adeno |  | Poor | T3 |  | N3 |  |
| 980063 | 49 | F | Adeno |  | Poor | T3 |  | N3 |  |
| 46404174 | 47 | F | Adeno | Diffuse | Poor | T2a | Infiltrative | N0 | G3 |
| 48982424 | 53 | F | Adeno | Diffuse | Poor | T2b | Infiltrative | N0 | G3 |
| 9432729 | 61 | F | Adeno |  | Mod | T3 |  | N2 |  |
| 76629543 | 92 | M | Adeno | Intestinal | Mod | T3 | Infiltrative | N0 | G2 |
| 6412929 | 73 | F | Adeno | Diffuse | Poor | T3 | Infiltrative | N0 | G3 |
| 5218170 | 66 | F | Adeno | Diffuse | Poor | T3 | Infiltrative | N2 | G3 |
| 66811693 | 68 | M | Adeno | Intestinal | Mod | T1 | Expansive | N1 | G2 |
| 82973565 | 69 | M | Adeno | Mixed | Poor | T3 | Infiltrative | N1 | G3 |
| 26109694 | 64 | M | Adeno | Diffuse | Poor | T2a | Infiltrative | N0 | G3 |
| 73291145 | 47 | M | Adeno | Intestinal | Well | T2b | Infiltrative | N1 | G1 |
| 61669256 | 56 | M | Adeno | Diffuse | Mod | T3 | Infiltrative | N2 | G2 |
| 15295992 | 67 | M | Adeno | Intestinal | Mod | T2a | Infiltrative | N0 | G2 |
| 57689477 | 68 | F | Adeno | Intestinal | Mod | T1 | Infiltrative | N0 | G2 |
| 87622942 | 56 | M | Adeno | Diffuse |  | T3 | Infiltrative | N2 |  |
| 65256293 | 65 | F | Adeno | Diffuse | Poor | T3 | Infiltrative | N3 | G3 |
| 96141474 | 75 | M | Adeno | Intestinal | Mod | T2b | Infiltrative | N1 |  |
| 91596276 | 68 | F | Adeno | Mixed | Poor | T3 | Infiltrative | N0 | G3 |
| 21080055 | 53 | M | Adeno | Intestinal | Poor | T2b | Infiltrative | N1 | G3 |
| 74765340 | 72 | F | Adeno | Intestinal | Mod | T2b | Infiltrative | N2 | G2 |
| 49375233 | 79 | F | Adeno | Intestinal | Mod | T3 | Infiltrative | N3 | G2 |
| 91228050 | 73 | M | Adeno | Mixed |  | T4 | Infiltrative | N2 |  |
| 20263644 | 63 | F | Adeno | Diffuse |  | T2b | Infiltrative | N1 |  |
| 58947266 | 80 | M | Adeno | Intestinal | Mod | T3 | Infiltrative | N1 | G2 |
| 32226415 | 77 | M | Adeno | Intestinal | Poor | T2b | Expansive | N1 | G3 |
| 38877042 | 65 | M | Adeno | Diffuse | Poor | T3 | Infiltrative | N1 | G3 |
| 47492137 | 62 | M | Adeno | Diffuse | Poor | T3 | Infiltrative | N2 | G3 |
| 31231321 | 71 | M | Adeno | Diffuse |  | T3 | Infiltrative | N3 |  |
| 63540193 | 72 | M | Adeno | Mixed | Mod | T2b | Infiltrative | N0 | G2 |
| 75554796 | 78 | F | Adeno | Intestinal | Mod | T3 | Infiltrative | N1 | G2 |
| 83507354 | 62 | M | Adeno | Intestinal | Poor | T3 | Infiltrative | N1 |  |
| 2000484 | 68 | M | Adeno | Intestinal | Mod | T3 |  | N1 |  |
| 2000521 | 67 | F | Adeno |  | Poor | T2b |  | N2 |  |
| 2000518 | 56 | M | Adeno | Diffuse | Poor | T2 |  | N0 |  |
| 2000529 | 77 | M | Adeno | Intestinal | Mod | T2 |  | N1 |  |
| 2000616 | 57 | M | Adeno |  | Poor | T2b |  | N1 |  |
| 2000617 | 77 | M | Adeno |  | Poor | T2b |  | N2 |  |
| 2000618 | 67 | M | Adeno |  | Mod | T2a |  | N1 |  |
| 2000619 | 69 | F | Adeno |  | Poor | T3 |  |  |  |
| 2000639 | 69 | M | Adeno | Intestinal | Mod | T2b |  | N2 |  |
| 2000676 | 85 | F | Adeno |  | Mod | T3 |  | N1 |  |
| 2000696 | 80 | F | Adeno |  | Poor | T2a |  | N0 |  |
| 2000708 | 58 | M | Adeno |  | Mod | T3 |  | N1 |  |
| 2000721 | 71 | M | Adeno |  | Poor | T3 |  | N2 |  |
| 2000732 | 60 | F | Adeno | Diffuse | Poor | T3 |  | N1 | G3 |
| 2000734 | 66 | M | Adeno |  | Poor | T4 |  | N1 |  |
| 2000752 | 61 | M | Adeno |  | Poor | T3 |  | N2 |  |
| 2000778 | 57 | M | Adeno | Mixed | Poor | T3 |  | N2 |  |
| 2000859 | 65 | M | Adeno |  | Poor | T3 |  | N3 |  |
| 2000877 | 45 | M | Adeno |  | Poor | T2a |  | N1 |  |
| 2000892 | 71 | F | Adeno | Intestinal |  | T2 |  | N1 |  |
| 2000920 | 80 | M | Adeno | Diffuse |  | T3 |  | N2 |  |
| 2000963 | 50 | M | Adeno |  |  | T2 |  |  |  |
| 2001086 | 80 | M | Adeno | Diffuse | Poor | T3 |  | N2 |  |
| 2001120 | 67 | M | Adeno |  | Mod | T3 |  | N2 |  |
| 2001123 | 56 | M | Adeno |  | Mod | T3 |  | N2 |  |
| 2001135 | 64 | M | Adeno |  | Poor | T3 |  | N0 |  |
| 2001159 | 78 | M | Adeno | Intestinal | Well | T1 |  | N0 |  |
| 2001190 |  |  | Adeno |  | Poor | T3 |  | N2 |  |
| 2001206 | 65 | M | Adeno | Diffuse | Poor | T3 |  | N2 | G3 |
| 990413 | 68 | M | Adeno |  | Poor | T3 |  | N1 |  |
| 990424 | 71 | M | Adeno | Mixed | Mod | T2b |  | N1 |  |
| 990475 | 71 | M | Adeno |  | Well | T2a |  | N1 |  |
| 990474 | 79 | F | Adeno | Intestinal | Poor | T3 |  | N0 |  |
| 990489 | 79 | M | Neuro |  |  | T2 |  | N0 |  |
| 990515 | 60 | M | Adeno |  | Mod | T3 |  | N2 |  |
| 2000040 | 70 | M | Adeno | Diffuse | Poor | T3 |  | N2 |  |
| 2000068 | 64 | F | Adeno |  | Mod | T1 |  | N0 |  |
| 2000088 | 53 | M | Adeno |  | Poor | T3 |  | N1 |  |
| 2000085 | 53 | M | Adeno | Intestinal | Mod | T3 |  | N0 |  |
| 2000114 | 53 | M | Adeno | Intestinal | Poor | T3 |  | N2 | G3 |
| 2000169 | 78 | M | Adeno |  | Poor | T3 |  | N2 |  |
| 2000175 | 64 | M | Adeno |  | Mod | T3 |  | N1 |  |
| 2000178 | 71 | F | Adeno |  | Poor | T2b |  | N1 |  |
| 2000201 | 70 | M | Adeno | Intestinal | Well | T2b |  | N0 |  |
| 2000238 | 74 | F | Adeno | Diffuse | Poor | T3 |  | N0 |  |
| 2000242 | 67 | F | Adeno | Intestinal | Mod | T2a |  | N0 |  |
| 2000256 |  |  | Adeno |  | Poor | T3 |  | N1 |  |
| 2000286 | 65 | M | Adeno |  | Mod | T3 |  | N0 |  |
| 2000291 | 68 | M | Adeno |  | Poor | T3 |  | N1 |  |
| 2000303 | 48 | M | Adeno |  | Poor | T3 |  | N1 |  |
| 2000346 | 32 | F | Adeno | Diffuse | Poor | T3 |  | N1 |  |
| 2000362 | 51 | M | Adeno |  | Mod | T3 |  | N2 |  |
| 2000403 | 68 | M | Adeno | Diffuse |  | T3 |  | N1 |  |
| 2000433 | 56 | F | Adeno |  |  |  |  |  |  |
| 2000434 | 67 | M | Adeno |  |  | T2 |  | N0 |  |
| 2000441 | 53 | M | Adeno |  | Poor | T3 |  | N2 |  |
| 2000472 | 82 | M | Adeno | Diffuse | Poor | T3 |  | N1 |  |
| 2000479 | 62 | M | Adeno | Intestinal | Mod | T3 |  | N2 |  |
| 990170 | 41 | F | Adeno |  | Poor | T3 |  | N1 |  |
| 990150 | 87 | F | Adeno | Diffuse | Poor | T2 |  | N1 |  |
| 990136 | 76 | M | Adeno |  | Mod | T1 |  | N0 |  |
| 990129 | 77 | F | Adeno |  | Mod | T3 |  | N1 |  |
| 990119 | 56 | M | Adeno |  | Poor | T3 |  | N1 |  |
| 990111 | 74 | F | Adeno |  | Mod | T1 |  | N0 |  |
| 990108 | 65 | M | Adeno | Intestinal | Mod | T2 |  | N1 |  |
| 990098 | 54 | F | Adeno |  | Poor | T3 |  | N2 |  |
| 990097 | 46 | M | Adeno | Intestinal | Poor | T3 |  | N2 |  |
| 990089 | 75 | M | Adeno |  | Mod | T3 |  | N2 |  |
| 990090 | 64 | M | Adeno | Intestinal | Mod | T3 |  | N1 |  |
| 990070 | 43 | M | Adeno |  | Poor | T3 |  | N1 |  |
| 990071 | 71 | F | Adeno |  | Mod | T3 |  | N0 |  |
| 990073 | 59 | M | Adeno |  | Mod | T2 |  | N2 |  |
| 990069 | 69 | M | Adeno | Diffuse | Poor | T2 |  | N0 |  |
| 990068 | 73 | M | Adeno | Diffuse | Poor | T3 |  | N1 |  |
| 990060 |  |  | Adeno | Diffuse | Poor | T3 |  | N2 |  |
| 990046 | 55 | M | Adeno |  | Mod | T3 |  | N2 |  |
| 990044 | 69 | M | Adeno |  | Mod | T3 |  | N2 |  |
| 990041 | 40 | M | Adeno | Intestinal | Mod | T3 |  | N1 |  |
| 990024 | 68 | M | Adeno |  | Mod | T3 |  | N1 |  |
| 990015 | 61 | M | Adeno |  | Mod | T1 |  | N0 |  |
| 990010 |  |  | Adeno |  | Poor | T3 |  | N2 |  |
| 990005 | 60 | M | Adeno |  | Poor | T3 |  | N2 |  |
| 980447 | 69 | M | Adeno |  | Poor | T3 |  | N2 |  |
| 980442 | 62 | F | Adeno |  | Poor | T3 |  | N1 |  |
| 980437 | 68 | F | Adeno |  | Poor | T3 |  | N2 |  |
| 980436 | 65 | F | Adeno | Intestinal | Mod | T3 |  | N1 |  |
| 980418 | 88 | M | Adeno |  | Mod | T3 |  | N1 |  |
| 980417 | 67 | M | Adeno | Intestinal | Poor | T3 |  | N2 |  |
| 980401 | 83 | F | Adeno | Diffuse | Poor | T3 |  | N1 |  |
| 980390 | 78 | F | Adeno |  | Mod | T1 |  | N1 |  |
| 980386 | 76 | F | Adeno | Diffuse | Poor | T3 |  | N2 |  |
| 980369 | 47 | F | Adeno |  | Poor | T2 |  | N0 |  |
| 980344 | 70 | F | Adeno |  | Poor |  |  | N1 |  |
| 980327 |  |  | Sq+Adeno |  | Poor | T3 |  | N1 |  |
| 980319 | 68 | M | Adeno |  | Poor | T3 |  | N1 |  |
| 990172 | 60 | M | Adeno | Intestinal | Mod | T2 |  | N2 |  |
| 990195 |  |  | Adeno |  | Mod | T3 |  | N1 |  |
| 990203 | 79 | M | Adeno |  | Poor | T3 |  | N1 |  |
| 990205 | 72 | F | Adeno | Diffuse | Poor | T2 |  | N1 |  |
| 990228 | 40 | F | Adeno |  | Poor | T3 | Signet | N1 |  |
| 990247 | 70 | M | Adeno |  | Poor | T3 |  | N0 |  |
| 990275 | 72 | M | Adeno | Intestinal | Well | T3 |  | N0 |  |
| 990300 | 50 | F | Adeno |  | Poor | T3 |  | N2 |  |
| 990399 |  |  | Adeno |  | Poor | T3 |  | N2 |  |
| 990355 | 34 | F | Adeno | Diffuse | Poor | T3 |  | N2 |  |
| 990396 | 65 | M | Adeno |  | Poor | T3 |  | N2 |  |
| 990412 |  |  | Adeno |  | Poor | T3 |  | N1 |  |
| 57701999 | 88 | M | Adeno | Intestinal | Mod | T3 | Tubular | N1 | G2 |
| 2001226 | 70 | M | Adeno | Diffuse | Poor | T3 | Signet | N2 |  |
| 2001229 | 49 | F | Adeno | Diffuse | Poor | T3 | Signet | N0 |  |
| 2001241 |  |  | Adeno | Diffuse | Poor | T3 | Signet | N0 |  |
| 20020011 | 49 | M | Adeno | Mixed | Poor | T3 | Signet | N1 | G3 |
| 20020032 | 56 | M | Adeno |  |  | T2b |  |  |  |
| 20020195 | 68 | M | Adeno |  | Poor | T3 |  | N1 |  |
| 20020361 | 63 | M | Adeno |  | Poor | T2 |  | N0 |  |
| 20020448 | 64 | M | Adeno |  | Poor | T2b |  | N2 | G3 |
| 20020455 | 81 | F | Adeno | Intestinal | Mod | T2 |  | N0 |  |
| 20020720 | 75 | M | Adeno | Intestinal | Mod | T2a | Tubular | N1 | G2 |
| 20020838 | 79 | M | Adeno | Intestinal | Mod | T2b | Tubular | N0 |  |
| 20020846 | 46 | M | Adeno | Diffuse | Poor | T2b | Tubular | N1 | G3 |
| 20020999 | 72 | F | Adeno | Diffuse | Poor | T2a | Tubular | N2 | G3 |
| 20021007 | 54 | M | Adeno |  | Poor | T2b |  | N0 |  |
| 20021146 | 71 | F | Adeno | Diffuse | Mod | T1is | Tubular | N0 |  |
| 10390127 | 27 | F | Adeno | Diffuse | Poor | T3 | Tubular | N0 | G3 |
| 68334421 | 64 | M | Adeno | Intestinal | Well | T2b | Papillary | N0 | G1 |
| 69245824 | 41 | F | Adeno | Diffuse | Poor | T3 | Signet | N3 | G4 |
| 47149013 | 77 | M | Adeno | Mixed |  | T2b | Signet | N3 |  |
| 98748381 | 66 | M | Adeno | Mixed | Poor | T3 | Signet | N2 | G3 |
| 73813499 | 68 | F | Adeno | Diffuse |  | T3 | Signet | N2 |  |
| 43658255 | 67 | M | Adeno | Intestinal | Mod | T3 | Tubular | N2 | G2 |
| 37262942 | 88 | F | Adeno | Diffuse | Poor | T2b | Tubular | N1 | G3 |
| 78373410 | 51 | F | Adeno | Diffuse |  | T3 | Signet | N3 |  |
| 31661621 | 84 | F | Adeno | Diffuse | Mod | T2b |  | N2 | G2 |
| 77263387 | 75 | M | Large Cell Neuroendocrine | |  | T2b |  | N1 |  |
| 29806547 | 66 | F | Adeno | Diffuse |  | T2b | Signet | N0 |  |
| 91515473 | 43 | M | Adeno | Mixed | Poor | T2b | Tubular | N2 | G3 |
| 7847924 | 64 | F | Adeno |  |  | T3 |  | N1 |  |
