# Supplementary material for: Oncogenic Pathway Combinations Predict Clinical Prognosis in Gastric Cancer
Source: PLoS Genet. 2009 Oct 2;5(10):e1000676. doi: 10.1371/journal.pgen.1000676 (PMC2748685; doi:10.1371/journal.pgen.1000676)
Supplement: Table S10 — Histopathological data for Cohort 3 of 31 tumors from the United Kingdom. (0.07 MB DOC) [file pgen.1000676.s014.doc]

Table S10. Histopathological data for Cohort 3 of 31 tumors from the United Kingdom

| **RNA ID** | **Age** | **Gender** | **Lauren** | **Differentiation** | **Stage** | **Ming** |
| --- | --- | --- | --- | --- | --- | --- |
| 3792 | 69.82 | male | diffuse | Poor | 3 | expansile |
| 3793 | 67.24 | male | intestinal | Moderate | 3 | infiltrative |
| 3794 | 76.39 | male | diffuse | Poor | 4 | . |
| 3798 | 56.85 | male | intestinal | Poor | 4 | infiltrative |
| 3810 | 63.75 | male | diffuse | Poor | 1 | infiltrative |
| 3811 | 83.73 | male | intestinal | Moderate | 2 | expansile |
| 3812 | 75.6 | female | diffuse | Poor | 3 | infiltrative |
| 3816 | 61.15 | male | intestinal | Moderate | 1 | expansile |
| 3817 | 72.17 | male | intestinal | Poor | 4 | expansile |
| 3820 | 71.41 | female | intestinal | Well | 1 | expansile |
| 3857 | 55.21 | male | mixed/unclassifiable | Poor | 3 | infiltrative |
| 3860 | 77.7 | female | intestinal | Moderate | 3 | infiltrative |
| 3861 | 73.31 | female | diffuse | Moderate | 4 | infiltrative |
| 3862 | 56.16 | male | intestinal | Moderate | 1 | infiltrative |
| 3863 | 53.04 | male | mixed/unclassifiable | Poor | 3 | infiltrative |
| 3864 | 74.74 | female | intestinal | Moderate | 3 | expansile |
| 3865 | 77 | male | intestinal | Moderate | 2 | expansile |
| 4352 | 74.57 | male | intestinal | Poor | 2 | infiltrative |
| 3796 | 59.19 | female | intestinal | Moderate | 3 | expansile |
| 3803 | 82.73 | female | intestinal | Moderate | 3 | expansile |
| 3813 | 76.59 | female | intestinal | Moderate | 3 | expansile |
| 3814 | 56.85 | male | intestinal | Poor | 4 | infiltrative |
| 3815 | 70.9 | male | intestinal | Poor | 3 | infiltrative |
| 3818 | 74.42 | male | mixed/unclassifiable | Poor | 2 | infiltrative |
| 3819 | 83.68 | female | diffuse | Poor | 3 | infiltrative |
| 3858 | 78.78 | male | intestinal | Well | 1 | expansile |
| 3859 | 78.55 | female | intestinal | Moderate | 3 | infiltrative |
| 4349 | 70.08 | female | intestinal | Moderate | 3 | infiltrative |
| 4350 | 83.09 | female | intestinal | Poor | 1 | expansile |
| 4351 | 81.52 | female | intestinal | Poor | 3 | infiltrative |
