# Supplementary material for: Oncogenic Pathway Combinations Predict Clinical Prognosis in Gastric Cancer
Source: PLoS Genet. 2009 Oct 2;5(10):e1000676. doi: 10.1371/journal.pgen.1000676 (PMC2748685; doi:10.1371/journal.pgen.1000676)
Supplement: Table S11 — Signatures associated with perturbed estrogen signaling. (0.03 MB DOC) [file pgen.1000676.s015.doc]

Table S11. Signatures associated with perturbed estrogen signaling.

| **Geneset ref.** | **Pathway** | **Description** | **Location of source** |
| --- | --- | --- | --- |
| Becker et al., 2005 [1] | Tamoxifen sensitivity | Estrogen-responsive genes which are downregulated in MaCa 3366/TAM compared to MaCa 3366 (fold change > 2) | http://www.broad.mit.edu/gsea/msigdb/cards/  BECKER_ESTROGEN_RESPONSIVE_SUBSET_2.html |
| Stossi et al., 2004 [2] | Estrogen response | Genes up-regulated by estradiol through ERalpha and ERbeta in U2OS cells | http://www.broad.mit.edu/gsea/msigdb/cards/  STOSSI_ER_UP.html |

**References**

1. Becker M, Sommer A, Krätzschmar JR, Seidel H, Pohlenz HD, et al. (2005) Distinct gene expression patterns in a tamoxifen-sensitive human mammary carcinoma xenograft and its tamoxifen-resistant subline MaCa 3366/TAM. Mol Cancer Ther 4: 151-168.

2. Stossi F, Barnett DH, Frasor J, Komm B, Lyttle CR, et al. (2004) Transcriptional profiling of estrogen-regulated gene expression via estrogen receptor (ER) alpha or ERbeta in human osteosarcoma cells: distinct and common target genes for these receptors. Endocrinology 145: 3473-3486.
