# Supplementary material for: Oncogenic Pathway Combinations Predict Clinical Prognosis in Gastric Cancer
Source: PLoS Genet. 2009 Oct 2;5(10):e1000676. doi: 10.1371/journal.pgen.1000676 (PMC2748685; doi:10.1371/journal.pgen.1000676)
Supplement: Table S12 — Signatures associated with 11 oncogenic pathways implicated in gastric carcinogenesis. (0.07 MB DOC) [file pgen.1000676.s016.doc]

Table S12. Signatures associated with 11 oncogenic pathways implicated in gastric carcinogenesis.

| **Geneset ref.** | **Pathway (context)** | **Description/Derivation** | **Location of source** |
| --- | --- | --- | --- |
| Assou et al., 2007 [1] | Stem cell (hESC) | Overexpressed in human embryonic stem cells compared to differentiated cell types in at least 10 studies | Table 3 in Assou et al., 2007 [1] |
| Ramalho-Santos et al., 2002 [2] | Stem cell (mESC) | Enriched in mouse embryonic stem cells, compared to differentiated brain and bone marrow cells | http://www.broad.mit.edu/gsea/msigdb/cards/  STEMCELL_EMBRYONIC_UP.html |
| Ramalho-Santos et al., 2002 [2] | Stem cell (mNSC) | Enriched in mouse neural stem cells, compared to differentiated brain and bone marrow cells | http://www.broad.mit.edu/gsea/msigdb/cards/  STEMCELL_NEURAL_UP.html |
| Menssen et al., 2002 [3] | MYC (umbilical) | Genes up-regulated by MYC in HUVEC (umbilical vein endothelial cell) | http://www.broad.mit.edu/gsea/msigdb/cards/  MENSSEN_MYC_UP.html |
| Bild et al., 2006 [4] | MYC (breast) | Regulated by infection with adenovirus expressing human c-Myc in human mammary epithelial cells | http://www.nature.com/nature/journal/v439/n7074/  extref/nature04296-s1.pdf |
| Stanelle et al., 2002 [5] | E2F (osteosarcoma) | Genes up-regulated by E2F1 in Saos2 (osteosarcoma) cells | http://www.broad.mit.edu/gsea/msigdb/cards/  STANELLE_E2F1_UP.html |
| Kalma et al., 2001 [6] | E2F (rat) | DNA replication genes upregulated by E2F1 induction in rat fibroblasts | http://www.broad.mit.edu/gsea/msigdb/cards/  E2F1_DNA_UP.html |
| Wu et al., 2002 [7] | p21-repressed | Down-regulated at any timepoint (4-24 hrs) following ectopic expression of p21 (CDKN1A) in OvCa cells, p53-dependent | http://www.broad.mit.edu/gsea/msigdb/cards/  P21_P53_ANY_DN.html |
| Hinata et al., 2003 [8] | NF-κB (skin) | Genes upregulated by NF-kappa B in epidermal keratinocytes and dermal fibroblasts | http://www.broad.mit.edu/gsea/msigdb/cards/  HINATA_NFKB_UP.html  http://www.broad.mit.edu/gsea/msigdb/cards/  HINATA_NFKB_DN.html |
| Tian et al., 2005 [9] | NF-κB (cervix) | Up-regulated at any timepoint following TNF-α treatment, only with functional NF-κB in HeLa cells | http://www.broad.mit.edu/gsea/msigdb/cards/  TNFA_NFKB_DEP_UP.html |
| Kannan et al., 2001 [10] | p53 (lung) | Target genes up regulated by p53 (lung cancer cell line) | http://www.broad.mit.edu/gsea/msigdb/cards/  KANNAN_P53_UP.html  http://www.broad.mit.edu/gsea/msigdb/cards/  KANNAN_P53_DN.html |
| Ongusaha et al., 2003 [11] | p53 (mouse) | Upregulated by expression of p53 in mouse embryonic fibroblasts | http://www.broad.mit.edu/gsea/msigdb/cards/  P53_BRCA1_UP.html |
| Bild et al., 2006 [4] | RAS | Regulated by infection with adenovirus expressing activated H-Ras in human mammary epithelial cells | http://www.nature.com/nature/journal/v439/n7074/  extref/nature04296-s1.pdf |
| Willert et al., 2002 [12] | Wnt | Genes up-regulated by Wnt-3A in NCCIT (teratocarcinoma) | http://www.broad.mit.edu/gsea/msigdb/cards/  WILLERT_WNT_NCCIT_ALL_UP.html |
| Welcsh et al., 2002 [13] | BRCA1 (kidney) | Upregulated by induction of exogenous BRCA1 in EcR-293 cells | http://www.broad.mit.edu/gsea/msigdb/cards/  BRCA1_OVEREXP_UP.html  http://www.broad.mit.edu/gsea/msigdb/cards/  BRCA1_OVEREXP_DN.html |
| Bae et al., 2004 [14] | BRCA1 (prostate) | Up-regulated with stable, ectopic overexpression of BRCA1 in DU-145 human prostate cancer cell lines, compared to neo-only controls | http://www.broad.mit.edu/gsea/msigdb/cards/  BRCA1_OVEREXP_PROSTATE_UP.html  http://www.broad.mit.edu/gsea/msigdb/cards/  BRCA1_OVEREXP_PROSTATE_DN.html |
| Bild et al., 2006 [4] | β-catenin | Regulated by infection with adenovirus expressing activated β-catenin in human mammary epithelial cells | http://www.nature.com/nature/journal/v439/n7074/  extref/nature04296-s1.pdf |
| Mariadason et al., 2000 [15] | HDAC inhibition (BUT) | Upregulated by butyrate at any timepoint up to 48 hrs in SW260 colon carcinoma cells | http://www.broad.mit.edu/gsea/msigdb/cards/  HDACI_COLON_BUT_UP.html  http://www.broad.mit.edu/gsea/msigdb/cards/  HDACI_COLON_BUT_DN.html |
| Mariadason et al., 2000 [15] | HDAC inhibition (TSA) | Upregulated by TSA at any timepoint up to 48 hrs in SW260 colon carcinoma cells | http://www.broad.mit.edu/gsea/msigdb/cards/  HDACI_COLON_TSA_UP.html  http://www.broad.mit.edu/gsea/msigdb/cards/  HDACI_COLON_TSA_DN.html |
| Bild et al., 2006 [4] | SRC | Regulated by infection with adenovirus expressing human c-Src in human mammary epithelial cells | http://www.nature.com/nature/journal/v439/n7074/  extref/nature04296-s1.pdf |

**References**

1. Assou S, Le Carrour T, Tondeur S, Ström S, Gabelle A, et al. (2007) A meta-analysis of human embryonic stem cells transcriptome integrated into a web-based expression atlas. Stem Cells 25.

2. Ramalho-Santos M, Yoon S, Matsuzaki Y, Mulligan RC, Melton DA (2002) "Stemness": transcriptional profiling of embryonic and adult stem cells. Science 298: 597-600.

3. Menssen A, Hermeking H (2002) Characterization of the c-MYC-regulated transcriptome by SAGE: identification and analysis of c-MYC target genes. Proc Natl Acad Sci U S A 99: 6274-6279.

4. Bild AH, Yao G, Chang JT, Wang Q, Potti A, et al. (2006) Oncogenic pathway signatures in human cancers as a guide to targeted therapies. Nature 439: 353-357.

5. Stanelle J, Stiewe T, Theseling CC, Peter M, Pützer BM (2002) Gene expression changes in response to E2F1 activation. Nucleic Acids Res 30: 1859-1867.

6. Kalma Y, Marash L, Lamed Y, Ginsberg D (2001) Expression analysis using DNA microarrays demonstrates that E2F-1 up-regulates expression of DNA replication genes including replication protein A2. Oncogene 20: 1379-1387.

7. Wu Q, Kirschmeier P, Hockenberry T, Yang TY, Brassard DL, et al. (2002) Transcriptional regulation during p21WAF1/CIP1-induced apoptosis in human ovarian cancer cells. J Biol Chem 277: 36329-36337.

8. Hinata K, Gervin AM, Zhang YJ, Khavari PA (2003) Divergent gene regulation and growth effects by NF-kappa B in epithelial and mesenchymal cells of human skin. Oncogene 22: 1955-1964.

9. Tian B, Nowak DE, Jamaluddin M, Wang S, Brasier AR (2005) Identification of direct genomic targets downstream of the nuclear factor-kappaB transcription factor mediating tumor necrosis factor signaling. J Biol Chem 280: 17435-17448.

10. Kannan K, Amariglio N, Rechavi G, Jakob-Hirsch J, Kela I, et al. (2001) DNA microarrays identification of primary and secondary target genes regulated by p53. Oncogene 20: 2225-2234.

11. Ongusaha PP, Ouchi T, Kim KT, Nytko E, Kwak JC, et al. (2003) BRCA1 shifts p53-mediated cellular outcomes towards irreversible growth arrest. Oncogene 22: 3749-3758.

12. Willert J, Epping M, Pollack JR, Brown PO, Nusse R (2002) A transcriptional response to Wnt protein in human embryonic carcinoma cells. BMC Dev Biol 2: 8.

13. Welcsh PL, Lee MK, Gonzalez-Hernandez RM, Black DJ, Mahadevappa M, et al. (2002) BRCA1 transcriptionally regulates genes involved in breast tumorigenesis. Proc Natl Acad Sci U S A 99: 7560-7565.

14. Bae I, Fan S, Meng Q, Rih JK, Kim HJ, et al. (2004) BRCA1 induces antioxidant gene expression and resistance to oxidative stress. Cancer Res 64: 7893-7909.

15. Mariadason JM, Corner GA, Augenlicht LH (2000) Genetic reprogramming in pathways of colonic cell maturation induced by short chain fatty acids: comparison with trichostatin A, sulindac, and curcumin and implications for chemoprevention of colon cancer. Cancer Res 60: 4561-4572.
