# Supplementary material for: Oncogenic Pathway Combinations Predict Clinical Prognosis in Gastric Cancer
Source: PLoS Genet. 2009 Oct 2;5(10):e1000676. doi: 10.1371/journal.pgen.1000676 (PMC2748685; doi:10.1371/journal.pgen.1000676)
Supplement: Text S1 — Supplementary methods. (0.05 MB DOC) [file pgen.1000676.s017.doc]

Oncogenic Pathway Combinations Predict Clinical Prognosis in Gastric Cancer

(Supplementary Methods)

Chia Huey Ooi^1^, Tatiana Ivanova^2^, Jeanie Wu^2^, Minghui Lee^2^, Iain Beehuat Tan^3^, Jiong Tao^2,4^, Lindsay Ward^5^, Jun Hao Koo^2^, Veena Gopalakrishnan^2^, Yansong Zhu^2^, Lai Ling Cheng^6^, Julian Lee^2^, Sun Young Rha^7^, Hyun Cheol Chung^7^, Kumaresan Ganesan^2^, Jimmy So^8^, Khee Chee Soo^9^, Dennis Lim^10^, Weng Hoong Chan^10^, Wai Keong Wong^10^, David Bowtell^11^, Khay Guan Yeoh^12^, Heike Grabsch^5^, Alex Boussioutas^11,13^, and Patrick Tan^1,2,14,15,*^

^1^[Duke-NUS Graduate Medical School,](http://www.gms.edu.sg/) [Singapore](http://www.gms.edu.sg/downloads/GMS_Map-240706_FINAL.pdf)

^2^Cellular and Molecular Research, National Cancer Centre, Singapore

^3^Division of Medical Oncology, National Cancer Centre, Singapore

^4^Department of Physiology, Yong Loo Lin School of Medicine, National University of Singapore, Singapore

^5^Section of Pathology and Tumour Biology, Leeds Institute of Molecular Medicine, St James's University Hospital, Leeds, United Kingdom

^6^Singapore-MIT Alliance, National University of Singapore, Singapore

^7^Department of Internal Medicine, Yonsei Cancer Center, Yonsei University College of Medicine, Seoul, Korea

^8^Department of Surgery, Yong Loo Lin School of Medicine, National University of Singapore, Singapore

^9^Division of Surgical Oncology, National Cancer Centre, Singapore

^10^Department of General Surgery, Singapore General Hospital, Singapore

^11^Cancer Genomics and Biochemistry Laboratory, Peter MacCallum Cancer Centre, East Melbourne, Victoria, Australia

^12^Department of Medicine, Yong Loo Lin School of Medicine, National University of Singapore, Singapore

^13^Department of Medicine (RMH/WH), University of Melbourne, Western Hospital, Footscray, Victoria, Australia

^14^Cancer Science Institute of Singapore, Yong Loo Lin School of Medicine, National University of Singapore, Singapore

^15^Genome Institute of Singapore, Singapore

Contact:

* Address correspondence to gmstanp@duke-nus.edu.sg

Tel : 65-6-436-8345

Fax : 65-6-226-5694

Running Title: Oncogenic Pathway Activity in Gastric Cancer

**Supplementary Methods**

**Data Preprocessing**

There are a total of 349 genome-wide mRNA profiles (33 from GC cell lines, 301 primary tumors, and 15 references). The GC cell line profiles, 270 of the primary tumor profiles, and the reference profiles (except reference profiles derived from the median of the cohort) were processed using HG-U133 Plus 2.0 arrays. The rest of the primary tumor profiles were processed using HG-U133A arrays. The scan data were converted to average difference values and confidence calls using Affymetrix’s Microarray Suite (MAS 5.0).

**Formation of Reference Profiles**

For primary gastric tumors and breast cancer cell lines, the median profile of the cohort was used as a reference profile. For gastric cancer cell lines (GCCLs), a total of seven reference profiles were used (Table S4). This was done exclusively for GCCLs in order to obtain high-confident candidate lines for *in vitro* models of specific pathway activations (example in Figure S2).

The median GCCL (GC cell line) profile was formed by computing the median expression values across the GCCL mRNA profiles. The CRL2072 reference profiles and the other five reference profiles (which represent different types of normal stomach tissue) were formed by computing the median of the multiple mRNA profiles associated with the reference. A probeset in the reference profile was given a ‘P’ confidence call if more than half of the confidence calls for the same probeset in the multiple mRNA profiles were ‘P’.

*Effect of Different References in GCCL Pathway Activation Profiles.* We made pairwise comparisons between pairs of different references by computing the Pearson correlation (Table S4) between activation scores obtained when generating the GCCL activation profiles for the 20 signatures listed in Error: Reference source not found. We also computed the p-value associated with each correlation. The associated p-value represents the probability of getting a correlation as large as the observed value by random chance, when the true correlation is zero.

**Sorting of Probesets in Cancer Profiles**

*Linear Scaling and Primary Thresholding.* Average difference values from gene expression profiles (of cancer cell lines or tumors) were scaled relative to the reference profile using a linear-fit-on-‘P’-call algorithm as described in Lamb et al. [1]. Average difference values less than a primary threshold value, *x*_min1_, were set to *x*_min1_. ($x_{\min1}=20$ for U133A profiles and $x_{\min1}=50$ U133 Plus 2.0 profiles.)

*Main Sorting and Secondary Thresholding*. For each probeset, the ratio of the average difference value in each cancer profile compared to the reference profile, *d*, was computed. Probesets were ranked in descending order of *d*. For probesets where $d=1$, a lower threshold, *x*_min2_, was applied to the average difference values, as described in Lamb et al. [1]. (We use $x_{\min2}=2$ for U133A profiles and $x_{\min2}=5$ for U133 Plus 2.0 profiles).

*Sub-sorting*. A new cancer to reference ratio, *d'*, was computed for probesets where $d=1$. These probesets were then sub-sorted in descending order of *d'*. Secondary thresholding and sub-sorting were applied to capture subtle differences in differential expression between cancer and reference profiles that might not be discernable in the main sorting.

**Reference**

1. Lamb J, Crawford ED, Peck D, Modell JW, Blat IC, et al. (2006) The Connectivity Map: using gene-expression signatures to connect small molecules, genes, and disease. Science 313(5795): 1929 - 1935.
